# Synthesis and *in Vitro* Antifungal Activity against *Botrytis cinerea* of Geranylated Phenols and Their Phenyl Acetate Derivatives

**DOI:** 10.3390/ijms160819130

**Published:** 2015-08-14

**Authors:** María I. Chávez, Mauricio Soto, Lautaro Taborga, Katy Díaz, Andrés F. Olea, Camila Bay, Hugo Peña-Cortés, Luis Espinoza

**Affiliations:** 1 Departamento de Química, Universidad Técnica Federico Santa María, Valparaíso 2340000, Chile; E-Mails: maria.chavez@usm.cl (M.I.C.); mauricio.sotoc.13@sansano.usm.cl (M.S.); lautaro.taborga@usm.cl (L.T.); katy.diaz@usm.cl (K.D.); 2 Instituto de Ciencias Químicas Aplicadas, Facultad de Ingeniería, Universidad Autónoma de Chile, Santiago 8910339, Chile; E-Mail: andres.olea@uautonoma.cl; 3 Facultad de Ingeniería, Universidad de Chile, Santiago 8370448, Chile; E-Mail: camila.bay.ch@gmail.com; 4 Facultad de Medicina, Hontaneda 2664, Universidad de Valparaíso, Valparaíso 2340000, Chile; E-Mail: hugo.pena@uv.cl

**Keywords:** *Botrytis cinerea*, antifungal activity, geranylphenols, synthesis, green chemistry

## Abstract

The inhibitory effects on the mycelial growth of plant pathogen *Botritys cinerea* have been evaluated for a series of geranylphenols substituted with one, two and three methoxy groups in the aromatic ring. The results show that the antifungal activity depends on the structure of the geranylphenols, increasing from 40% to 90% by increasing the number of methoxy groups. On the other hand, the acetylation of the –OH group induces a change of activity that depends on the number of methoxy groups. The biological activity of digeranyl derivatives is lower than that exhibited by the respective monogeranyl compound. All tested geranylphenols have been synthesized by direct coupling of geraniol and the respective phenol. The effect of solvent on yields and product distribution is discussed. For monomethoxyphenols the reaction gives better yields when acetonitrile is used as a solvent and AgNO_3_ is used as a secondary catalyst. However, for di- and trimethoxyphenols the reaction proceeds only in dioxane.

## 1. Introduction

**Figure 1:**
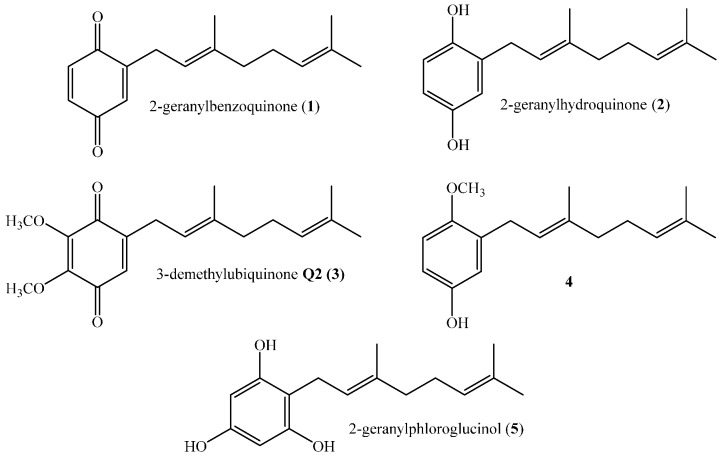
Structures of some active linear geranylphenols.

Prenylated compounds with one or more prenyl moieties are common natural products that have been isolated predominantly from marine organisms and plants [1,2,3,4]. These compounds have attracted much attention because they often possess antimicrobial, antioxidant, anti-inflammatory, antiviral and anticancer activities [5,6,7,8]. Various attempts to establish structure–activity relationships (SARs) relating to these biological activities with structural features of metabolites and their derivatives have been made [1,6]. The results indicate that these activities depend on the length of the side chain and the nature and relative position of substituent groups on the aromatic ring. The most active compounds are those having two isoprene units in the prenyl chain, and the chemical structures of some active geranylphenols are shown in Figure 1.

Based on these antecedents, we have undertaken a study of the effect of a series of geranylphenols on the mycelial growth inhibition of plant pathogen *Botrytis cinerea* as a function of molecular structure [9]. This knowledge might be useful to design and synthesize new substances to control such kinds of pathogens. Thus, the purpose of this work is to increase the comprehension of the structural features determining the antifungal activity of these compounds. With this aim in mind, the synthesis, structure determination, and inhibitory activity of a series of 15 geranylphenols with different numbers of methoxy groups in the aromatic ring are reported in this work.

The effect of these compounds on the mycelial growth of plant pathogen *B. cinerea* has been evaluated and the results are discussed in terms of their molecular structure. The effect of the structure of the starting phenol over the yield and nature of products obtained with both methods is also assessed.

## 2. Results and Discussion

**Figure 2:**
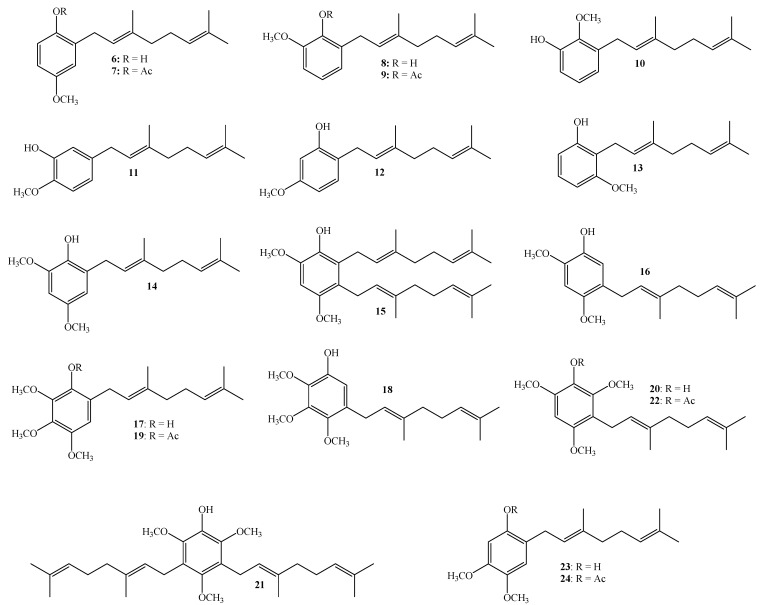
Structures of linear geranylphenols **6**–**24** tested in this work.

Compounds **6**–**24** (see Figure 2) were synthesized through the electrophilic aromatic substitution reaction (EAS), which proceeds by the slow addition of geraniol to a solution of phenol derivatives in dioxane and in the presence of BF_3_·Et_2_O which acts as catalyst [1,6,10,11]. This reaction was chosen, between many different synthetic pathways for obtaining prenylated compounds [1,11,12,13,14], because it has been previously used to synthesize a series of synthetic analogs of 3-demethylubiquinone (**3**) having one, two, or three methoxy groups in different positions of the quinone moiety [6]. Alternatively, the reaction was also carried out in acetonitrile instead of dioxane, using AgNO_3_ as a secondary catalyst [9]. The results indicate that both yields and products depend on the solvent.

### 2.1. Synthesis

**Scheme 1:**
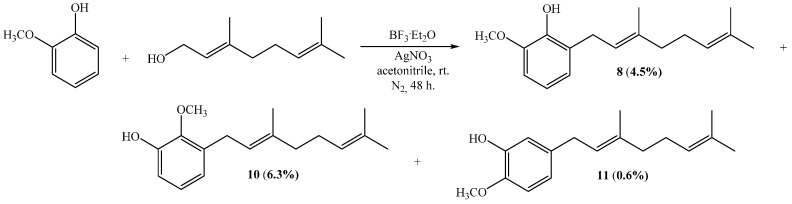
Synthesis of compounds **8**, **10** and **11**.

The direct coupling between geraniol and commercially available 4-methoxyphenol using acetonitrile/AgNO_3_ gives compound **6** as a unique product with 18% yield, which is almost twice the yield reported for the same reaction in dioxane (10%) [15]. However, this yield is much lower than that reported for the reaction of 4-methoxyphenol with geranyl bromide in the presence of NaH, which leads to **6** with a 56% yield [16]. On the other hand, the reaction between geraniol and guaiacol (2-methoxyphenol) generates compounds **8**, **10** and **11** according to Scheme 1. The yields of **8**, **10** and **11** in acetonitrile/AgNO_3_ were 4.5%, 6.3% and 0.6%, respectively, which are relatively higher than those obtained using dioxane, *i.e.*, 2.5%, 4.1% and 0.3%, respectively [11].

**Scheme 2:**
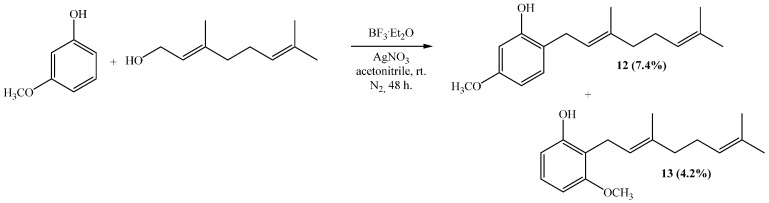
Synthesis of compounds **12** and **13**.

Compounds **12** and **13** were obtained by the coupling of geraniol and 3-methoxyphenol (3-hydroxyanisol) in acetonitrile, with yields of 7.4% and 4.2%, respectively (Scheme 2). Both substances are new compounds and their structure characterization is described in the next section.

Thus, the geranylation of monomethoxy phenols is more efficient when it is carried out in the presence of acetonitrile/AgNO_3_, with yields depending on the position of the methoxy substituent group. The reaction yield follows the order: para > metha ≈ ortho. This result can be explained in terms of ring activation by hydroxyl and methoxy groups, which is higher for the 4-methoxyphenol.

The acetylated derivatives **7** and **9** were obtained by a standard acetylation reaction of **6** and **8** with 95% and 93% yields, respectively. The NMR data of compounds **6**–**8**, **10** and **11** were consistent with those previously reported [11], whereas the NMR of compound **9** confirm the presence of the acetylated derivative (see below).

**Scheme 3:**
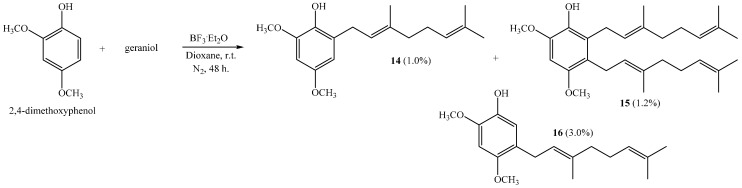
Preparation of dimethoxygeranylphenols **14**–**16**.

The coupling reaction between 2,4-dimethoxyphenol and geraniol leads to compounds **14**, **15** and **16**, with 1.0%, 1.2% and 3.0% yields, respectively (Scheme 3), when dioxane is used as a solvent.

Interestingly, a digeranylphenol **15** is obtained with very low yield. The coupling of two prenyl chains has been described for the reactions of 2,3,4-trimethoxyphenol with geraniol; 2,4-dimethoxyphenol with farnesol; and 1,3,5-trihydroxybenzene with geraniol [6,11]. In acetonitrile there is no coupling reaction, whereas compound **14** is the only product when ether is the solvent [6]. This compound is obtained with 46% by a reaction of 2,4-dimethoxyphenol and geranyl bromide [17]. The NMR data of compounds **14** and **16** were consistent with those previously reported [6,10].

The coupling reaction between trimethoxyphenols and geraniol follows a more complex pattern than that exhibited by dimethoxyphenols [6,10]. For all of them, the coupling reaction using dioxane as a solvent leads to different products depending on the phenol structure, whereas in the presence of acetonitrile/AgNO_3_ there is no reaction (see Table 1). Three isomers of trimethoxyphenol, namely 2,3,4-trimethoxyphenol, 2,4,5-trimethoxyphenol and 2,4,6-trimethoxyphenol, were synthesized by Baeyer-Villiger oxidation of the respective trimethoxybenzaldehyde, which was commercially available, and the subsequent saponification of the resulting trimethoxyphenyl formate [10].

**Scheme 4:**
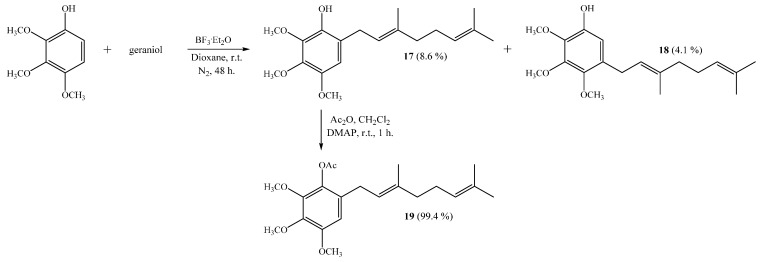
Synthesis of compounds **17**–**18** by the coupling reaction of 2,3,4-trimethoxyphenol with geraniol in dioxane. Compound **19** was obtained by acetylation of **17**.

The coupling of 2,3,4-trimethoxyphenol with geraniol leads to monogeranyl compounds **17** and **18** with 8.6% and 4.1% yields, respectively (Scheme 4). Subsequently, compound **19** was obtained from **17** with a 99.4% yield by standard acetylation. The NMR data of compound **17** were consistent with those previously reported [6].

**Scheme 5:**
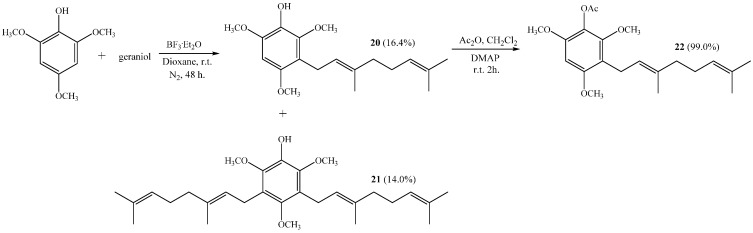
Synthesis of geranylmethoxyphenols **20**, **21** and acetylated derivative **22**.

The reaction of 2,4,6-trimethoxyphenol with geraniol gives compound **20** and with digeranyl gives compound **21** with 16.4% and 14.0% yields, respectively (see Scheme 5). Both compounds are new and their chemical structures were determined by NMR (see structure determination section).

Compound **22** was obtained from **20** with a 99.0% yield by standard acetylation.

**Scheme 6:**
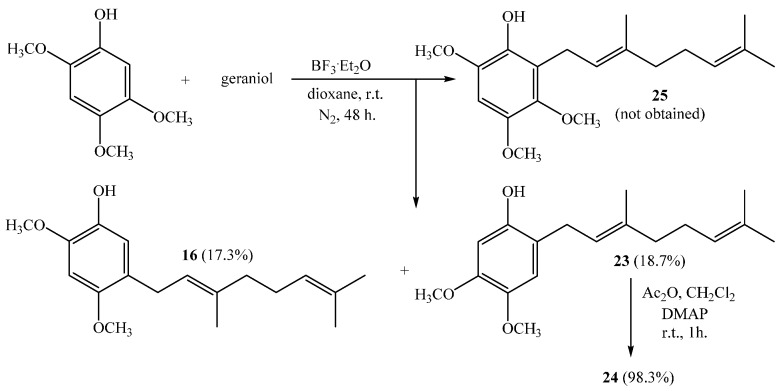
Obtaining of geranylmethoxyphenols **16** and **23** and acetylated derivative **24**.

Finally, the direct geranylation of 2,4,5-trimethoxyphenol leads unexpectedly to compounds **16** and **23** with 17.3% and 18.7% yields, respectively, whereas the mono-geranylated compound **25** was not obtained (see Scheme 6).

The formation of these compounds has been previously reported by our research group with slightly lower yields. It has been explained in terms of a competing reaction occurring via formation of a geranyl-ether intermediate, a subsequent Claisen rearrangement, and loss of a formaldehyde molecule from the methoxyl group [10]. The NMR data for both (**16** and **23**) compounds were consistent with those previously reported [10].

Finally, compound **24** was obtained from **23** with a 98.3% yield by standard acetylation.

**Table 1:** Synthesis conditions and yield reactions of geranylated phenols and phenyl acetate derivatives.

Compound	Reactants	Catalyst	Solvent	Yield
**6**	4-methoxyphenol/geraniol	BF_3_·Et_2_O/AgNO_3_	Acetonitrile	18.0
**8**	2-methoxyphenol/geraniol	BF_3_·Et_2_O/AgNO_3_	Acetonitrile	4.5
**10**	2-methoxyphenol/geraniol	BF_3_·Et_2_O/AgNO_3_	Acetonitrile	6.3
**11**	2-methoxyphenol/geraniol	BF_3_·Et_2_O/AgNO_3_	Acetonitrile	0.6
**12**	3-methoxyphenol/geraniol	BF_3_·Et_2_O/AgNO_3_	Acetonitrile	7.4
**13**	3-methoxyphenol/geraniol	BF_3_·Et_2_O/AgNO_3_	Acetonitrile	4.2
**14**	2,4-dimethoxyphenol/geraniol	BF_3_·Et_2_O	Dioxane	1.0
**15**	2,4-dimethoxyphenol/geraniol	BF_3_·Et_2_O	Dioxane	1.2
**16**	2,4,5-trimethoxyphenol/geraniol	BF_3_·Et_2_O	Dioxane	17.3
**17**	2,3,4-trimethoxyphenol/geraniol	BF_3_·Et_2_O	Dioxane	8.6
**18**	2,3,4-trimethoxyphenol/geraniol	BF_3_·Et_2_O	Dioxane	4.1
**20**	2,4,6-trimethoxyphenol/geraniol	BF_3_·Et_2_O	Dioxane	16.4
**21**	2,4,6-trimethoxyphenol/geraniol	BF_3_·Et_2_O	Dioxane	14.0
**23**	2,4,5-trimethoxyphenol/geraniol	BF_3_·Et_2_O	Dioxane	18.7

### 2.2. Structure Determination

**Figure 3:**
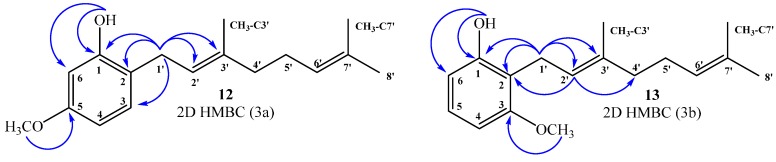
Most important correlations 2D ^1^H–^13^C HMBC, compound **12** (**3a**) and compound **13** (**3b**).

The chemical structure of all new compounds obtained through the synthesis described above was mainly established by NMR spectroscopy. All NMR spectra are given as Supplementary Material (Figure S1). Thus, in this section, the NMR data used to determine the chemical structure of geranylphenol derivatives (**12**–**13**, **15**, **18**, **20**–**21**) and acetylated derivatives (**9**, **19**, **22**, **24**) is discussed in detail. Compounds **12**–**13**: The ^1^H NMR spectrum of compound **12** shows a pattern characteristic of aromatic tri-substitution, *i.e.*, doublet signal at 6.99 ppm (*J* = 8.0 Hz, 1H, H-3); double doublet at 6.44 ppm (*J* = 2.5 and 8.0 Hz, 1H, H-4); and doublet at 6.42 ppm (*J* = 2.5 Hz, 1H, H-6). The position of the geranyl chain on the aromatic ring was established by two-dimensional (2D) HMBC correlations. In this spectrum, a ^2^*J*_H–C_ coupling of H-1ʹ with C-2 (δ_C_ = 118.9 ppm) and C-2ʹ (δ_C_ = 122.0 ppm), and a ^3^*J*_H–C_ coupling between the signals of C-1, C-3 and C-3ʹ at δ_C_ = 155.4, 130.3 and 138.4 ppm, respectively, were observed. In addition, a correlation at ^3^*J*_H–C_ between the CH_3_O group (δ_H_ = 3.76 ppm) and C-5 (δ_C_ = 159.4 ppm), and correlations between ^2^*J*_H–C_ and ^3^*J*_H–C_ of the OH group (δ_H_ = 5.25 ppm) with C-1 (δ_C_ = 155.4 ppm) and C-6 (δ_C_ = 102.0 ppm) were also observed (Figure 3a).

Similarly, for compound **13**, the ^1^H NMR spectrum shows a double doublet signal at 7.06 ppm (*J* = 8.2 and 8.2 Hz, 1H, H-5) and a doublet signal at 6.49 ppm (*J* = 8.2 Hz, 2H, H-4 and H-6). The aromatic substitution pattern shows unequivocally that the geranyl chain is attached to the *ortho* position between hydroxyl and methoxyl groups. The position of the geranyl chain on the aromatic ring has been confirmed by 2D HMBC correlations. In this spectrum, the signal at δ_H_ = 3.43 ppm assigned to H-1ʹ (d, *J* = 7.0 Hz, 2H) shows ^3^*J*_H–C_ coupling with C-1 (δ_C_ = 155.6) and C-3ʹ (δ_C_ = 138.1 ppm), and ^2^*J*_H–C_ coupling with C-2 and C-2ʹ (δ_C_ = 115.2 and 121.9 ppm, respectively), while the signal at δ_H_ = 5.24 ppm assigned to H-2ʹ (t, *J* = 7.0 Hz, 1H) shows ^3^*J*_H–C_ coupling with C-2 (δ_C_ = 115.2) and C-4ʹ (δ_C_ = 39.7 ppm). Additionally, the proton signal of the hydroxyl group (δ_H_ = 5.32 ppm) shows ^2^*J*_H–C_ coupling with C-1 (δ_C_ = 155.6 ppm) and ^3^*J*_H–C_ with C-6 (δ_C_ = 108.1 ppm). The correlation at ^3^*J*_H–C_ between the CH_3_O group (δ_H_ = 3.81 ppm) and C-3 (δ_C_ = 157.9 ppm) was also observed (Figure 3b).

**Figure 4:**
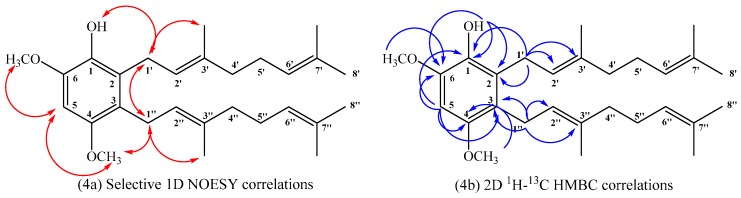
Main correlations observed for compound **15**, selective 1D NOESY (**4a**), and 2D^1^H–^13^C HMBC (**4b**).

Compound **15**: In the ^1^H NMR spectrum a single hydrogen aromatic signal was observed at δ_H_ = 6.42 ppm (s, 1H), confirming the aromatic disubstitution pattern. The positions of geranyl chains on the aromatic ring were established by selective one-dimensional (1D) NOESY experiments, wherein the aromatic signal at δ_H_ = 6.42 ppm assigned to H-5 showed spatial correlations with two singlets at 3.87 and 3.77 ppm, assigned to CH_3_O-C6 and CH_3_O-C4 (by 2D HSQC and HMBC), respectively. In addition, the signal at δ_H_ = 3.39 ppm (d, *J* = 6.0 Hz, 2H) assigned to hydrogen H-1ʹ showed spatial correlations with the signals at δ_H_ = 5.31 (s, 1H) and δ_H_ = 1.76 ppm (s, 3H), assigned to OH and CH_3_-C3ʹ, respectively. The signal at δ_H_ = 3.31 ppm (d, *J* = 6.0 Hz, 2H) assigned to hydrogen H-1ʹʹ showed spatial correlations with CH_3_O-C4 and with δ_H_ = 1.74 ppm (s, 3H) assigned to CH_3_-C3ʹʹ. Ortho aromatic disubstitution of both geranyl chains was confirmed by the observation of spatial NOE interaction between hydrogen H-1ʹ and H-1ʹʹ (see Figure 4a). Finally, the complete structural determination was established by heteronuclear 2D HSQC and 2D HMBC correlations, where H-1ʹ showed ^3^*J*_H–C_ coupling with C-3 (δ_C_ = 122.1 ppm), C-3ʹ (δ_C_ = 135.2 ppm) and C-1 (δ_C_ = 137.9 ppm) and ^2^*J*_H–C_ coupling with C-2 and C-2ʹ (δ_C_ = 127.3 and 123.8 ppm, respectively). H-1ʹʹ showed ^3^*J*_H–C_ coupling with C-4 (δ_C_ = 150.7 ppm), C-3ʹʹ (δ_C_ = 134.4 ppm) and C-2 (δ_C_ = 127.3 ppm) and ^2^*J*_H–C_ coupling with C-2ʹʹ and C-3 (δ_C_ = 122.8 and 122.1 ppm, respectively). The signal of H-5 at δ_H_ = 6.42 ppm (s, 1H-Ar) showed^3^*J*_H–C_ coupling with C-3 and C-1 (δ_C_ = 122.1 and 137.9 ppm, respectively) and ^2^*J*_H–C_ coupling with C-6 and C-4 (δ_C_ = 144.4 and 150.7 ppm, respectively). These and other heteronuclear correlations are shown in Figure 4b.

**Figure 5:**
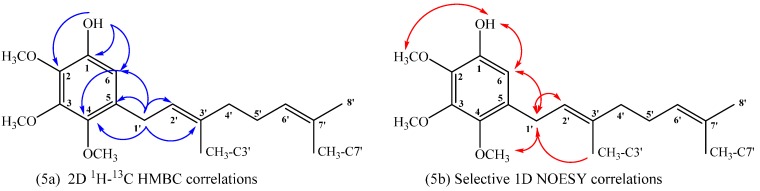
Main correlations observed for compound **18**, 2D ^1^H–^13^C HMBC (**5a**), selective 1D NOESY (**5b**).

Compound **18**: The ^1^H NMR of compound **18** shows aromatic signal at δ_H_ = 6.50 ppm (s, 1H) confirming the aromatic monosubstitution. On the other hand, in the HMBC spectrum, the signal at δ_H_ = 3.27 ppm, assigned to H-1ʹ (d, *J* = 7.2 Hz, 2H), shows ^3^*J*_H–C_ coupling with C-6 (δ_C_ = 109.5 ppm), C-3ʹ (δ_C_ = 136.2 ppm) and C-4 (δ_C_ = 144.9 ppm) and ^2^*J*_H–C_ coupling with C-2ʹ and C-5 (δ_C_ = 122.5 and 130.6 ppm, respectively). While the signal at δ_H_ = 6.50 ppm, assigned to H-6 (s, 1H), shows ^3^*J*_H–C_ coupling with C-1ʹ (δ_C_ = 27.8), C-2 (δ_C_ = 138.0 ppm) and C-4 (δ_C_ = 144.9 ppm) and the signal at δ_H_ = 5.46 ppm, assigned to OH (s, 1H), shows ^3^*J*_H–C_ coupling with C-6 (δ_C_ = 109.5 ppm) and C-2 (δ_C_ = 138.0 ppm) and ^2^*J*_H–C_ coupling with C-1 (δ_C_ = 144.5 ppm). These HMBC correlations are shown in Figure 5a. Finally, selective 1D NOESY NMR experiments were recorded for compound **18**. These correlations are shown in Figure 5b, where the most important ones correspond to the correlations observed between H-6 with OH and H-1ʹ at (5.46 and 3.27 ppm, respectively) and correlations between H-1ʹ with CH_3_O-C4 and CH_3_-C3ʹ (3.78 and 1.70 ppm, respectively).

**Figure 6:**
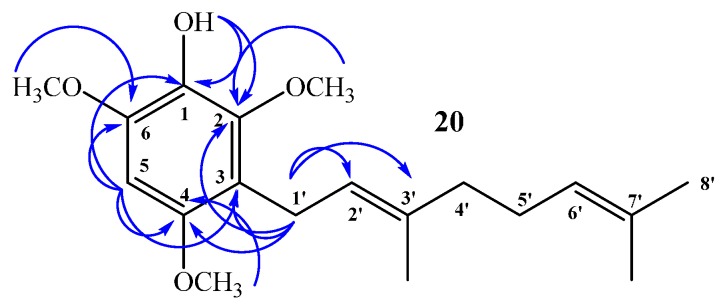
Main correlations 2D ^1^H–^13^C HMBC for compound **20**.

Compound **20**: In the ^1^H NMR spectrum a single signal at δ_H_ = 6.32 ppm (s, 1H) confirms aromatic monosubstitution. Unequivocally, there is only one possibility of aromatic monosubstitution and this is reflected in the HMBC spectrum, *i.e.*, the signal at δ_H_ = 3.30 ppm, assigned to H-1ʹ (d, *J* = 6.8 Hz, 2H), correlated with δ_C_ = 22.5 ppm (by 2D HSQC) and shows ^3^*J*_H–C_ coupling with C-2 (δ_C_ = 145.9 ppm), C-4 (δ_C_ = 150.6 ppm) and with C-3ʹ (δ_C_ = 134.4 ppm) and ^2^*J*_H–C_ coupling with C-3 and C-2ʹ (δ_C_ = 116.8 and 123.5 ppm, respectively). The signal at δ_H_ = 6.32 ppm, assigned to H-5 (s, 1H, by 2D HSQC), shows ^3^*J*_H–C_ coupling with C-3 (δ_C_ = 116.8 ppm) and C-1 (δ_C_ = 133.0 ppm), and ^2^*J*_H–C_ coupling with C-2 (δ_C_ = 145.9 ppm) and C-4 (δ_C_ = 150.6 ppm). In addition, the signal at δ_H_ = 5.15 ppm, assigned to OH (s, 1H), shows ^3^*J*_H–C_ coupling with C-6 (δ_C_ = 145.3 ppm) and C-2 (δ_C_ = 145.9 ppm), and ^2^*J*_H–C_ coupling with C-1 (δ_C_ = 133.0 ppm). These and other HMBC correlations are shown in Figure 6.

**Figure 7:**
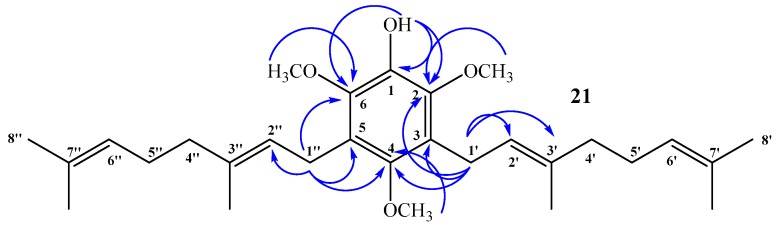
Most important 2D ^1^H–^13^C HMBC correlations observed for compound **21**.

Compound **21**: No aromatic hydrogen signals were detected in the ^1^H NMR spectrum, whereas a 1:2 ratio of integration areas was observed between the signal at δ_H_ = 3.66 ppm, assigned to CH_3_O-C4 (by 2D HSQC), and the signal at δ_H_ = 3.81 ppm, assigned to CH_3_O-C2 + CH_3_O-C6 (by 2D HSQC), confirming that this is a symmetrical disubstituted aromatic compound. The complete structure determination of compound **21** was established mainly by 2D HSQC and 2D HMBC experiments. The signal at δ_H_ = 3.34 ppm (d, *J* = 6.6 Hz, 4H), assigned to hydrogen atoms H-1ʹ and H-1ʹʹ, correlated with δ_C_ = 23.5 ppm (by 2D HSQC) and shows ^3^*J*_H–C_ coupling with C-2 and C-6 (δ_C_ = 144.4 ppm, 2 × C), respectively, and with C-4 (δ_C_ = 149.5 ppm). In addition, ^3^*J*_H–C_ coupling with C-3ʹ and C-3ʹʹ (δ_C_ = 134.8 ppm, 2 × C), and ^2^*J*_H–C_ coupling with C-3, C-5 (δ_C_ = 124.4 ppm, 2 × C), and with C-2ʹ and C-2ʹʹ (δ_C_ = 123.8 ppm, 2 × C). The signal at δ_H_ = 5.34 ppm, assigned to OH (s, 1H), shows ^3^*J*_H–C_ coupling with C-2 and C-6 (δ_C_ = 144.4 ppm, 2 × C) and ^2^*J*_H–C_ coupling with C-1 (δ_C_ = 139.0 ppm). These and other HMBC correlations are shown in Figure 7.

Phenyl acetate derivative compounds **9**, **19**, **22** and **24**: In the ^1^H NMR spectrum of these compounds, a signal at δ_H_ = 2.32 ppm (s, 3H, CH_3_CO) is observed, whereas signals at δ_C_ = 168.9 ppm (CH_3_CO) and δ_C_ = 20.5 ppm (CH_3_CO) in the ^13^C NMR spectrum are found. This confirms the presence of the acetylated derivative.

### 2.3. Antifungal Activity against B. cinerea in Vitro

**Figure 8:**
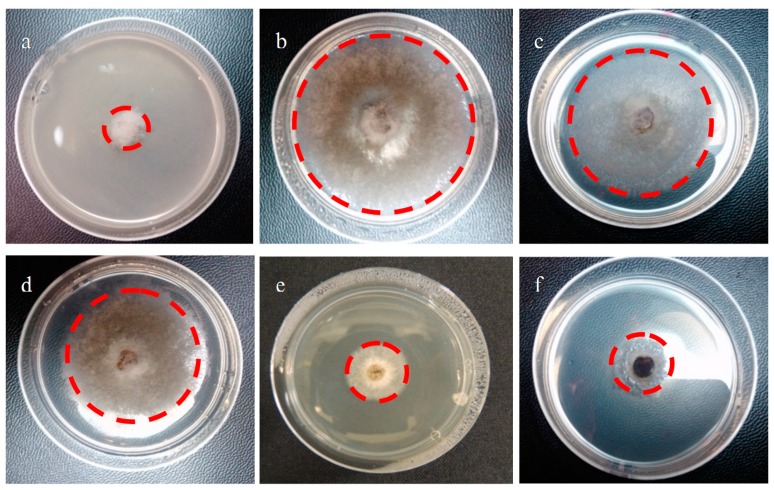
Effect of geranylated phenol/phenyl acetate derivatives on *in vitro* mycelial growth of *B. cinerea*. (**a**) Positive control, Captan; (**b**) negative control, the medium contains only PDA and 1% ethanol; (**c**) compound **15**; (**d**) compound **6**; (**e**) compound **9**; (**f**) compound **17**. Concentration of Captan and all tested compounds is 250 mg/L. The red line gives the growth radius of the negative control (**b**) and in presence of different compounds.

The effect of compounds **6**–**24** (Figure 2) on the mycelial growth of plant pathogen *B. cinerea* was evaluated *in vitro* after 48 h of incubation by using the agar-radial test with potato dextrose agar (PDA) as a medium. Figure 8 shows an assay where the mycelium of *B. cinerea* grows in the presence of Captan (Figure 8a, commercial fungicide widely applied to control diseases of fruits and vegetable crops and used in this study as a positive control) or of different geranylphenol compounds (Figure 8c–Figure 8f). Figure 8b corresponds to the negative control, *i.e.*, no inhibitory compounds. As it can be seen, Captan and compounds **9** and **17** almost completely inhibit the fungus growth, while compounds **15** and **6** are less active at a concentration of 250 mg/L.

The effect on mycelial growth is evaluated by comparing the growth areas with that observed for the negative control. The results are expressed as a percentage of inhibition, which is calculated as the ratio of the area of *B. cinerea* in the presence and absence of geranylated phenol/phenyl acetate derivatives, and they are summarized in Table 2.

The data in Table 2 indicates that for most of the studied compounds the percentage of inhibition increases with the increasing compound concentration, reaching maximum values that are between 53% and 98%. For compounds **6**, **15**, **19** and **20**, the effect levels off at 150 mg/L, and compounds **6**, **8**, **10**, **12**, **15** and **22** reduce growth in a quantity lower than 50%. On the other hand, the monomethoxygeranyl acetates **7** and **9** and trimethoxygeranylphenols **17** and **18** exhibit an inhibitory effect on mycelial growth similar to that found for Captan, *i.e.*, 86% to 98% at 250 mg/L. In a previous work, we have found that the inhibitory effect of Captan changed more deeply with concentration, *i.e.*, 40%, 60% and 80% at 50, 150 and 250 mg/L, respectively [9]. Both sets of experiments were carried out under the same conditions and, therefore, this remarkable difference is difficult to explain. It could be attributed to seasonal changes in the growing rate of *B. cinerea* or changes in the concentration of the commercial fungicide. However, as Captan is used only for comparison, the following discussion of the antifungal activity of geranylphenols as a function of chemical structure is not affected by this change.

**Table 2:** Effect of geranylated phenol/phenyl acetate derivatives on *in vitro* mycelial growth of *B. cinerea*, measured as a percentage of inhibition.

Geranylated Phenols/Phenyl Acetates Derivatives	Percentage of Inhibition on *in Vitro* Mycelial Growth of *B. cinerea* (%) *		
	50 mg/L	150 mg/L	250 mg/L
**6**	24 ± 4.6	42 ± 4.5	40 ± 4.8
**7**	5 ± 1.9	56 ± 5.3	86 ± 7.2
**8**	18 ± 3.5	31 ± 3.4	41 ± 4.6
**9**	13 ± 2.8	79 ± 8.7	98 ± 1.4
**10**	0	4 ± 1.4	26 ± 5.4
**12**	5 ± 1.0	20 ± 4.8	45 ± 4.0
**14**	9 ± 2.1	46 ± 2.8	54 ± 4.8
**15**	0	20 ± 2.0	21 ± 1.9
**16**	32 ± 2.2	41 ± 4.7	71 ± 3.8
**17**	13 ± 2.6	39 ± 4.0	91 ± 0.7
**18**	0	66 ± 6.4	91 ± 0.7
**19**	36 ± 6.6	55 ± 6.1	56 ± 4.6
**20**	18 ± 2.9	67 ± 5.5	73 ± 3.1
**21**	1 ± 0.4	20 ± 4.2	66 ± 3.9
**22**	4 ± 1.4	18 ± 3.0	39 ± 4.5
**23**	49 ± 3.3	59 ± 3.0	79 ± 2.1
**24**	42 ± 4.1	57 ± 3.0	77 ± 3.3
C−	0	0	0
C+	89 ± 2.1	94 ± 1.1	94 ± 1.3

* The percentage of inhibition of mycelial growth was based on colony diameter measurements after 72 h of incubation. Each point represents the mean of at least three independent experiments ± standard deviation.

The dependence of antifungal activity on chemical structure is more clearly understood by separating the studied compounds in structurally related groups. In the first place, the results obtained for monogeranyl compounds show that the activity depends clearly on the number of methoxy groups. The inhibitory effect of monomethoxyphenol compounds is so low that they are considered inactive, but when the number of methoxy groups is increased from one to three, the percentage of inhibition increases by a factor of two, reaching values similar to that measured for Captan. The order of ascending activity is monomethoxy (**6**, **8**, **10**, **12**) < dimethoxy (**14**, **16**, **23**) < trimethoxy (**17**, **18**, **22**). In the case of acetylated derivatives this effect is the opposite, *i.e.*, the activity decreases with the increasing number of methoxy groups and the order is monomethoxy (**7** and **9**) > dimethoxy (**24**) > trimethoxy (**19** and **22**). Interestingly, the acetylation of the hydroxyl group in monomethoxy compounds increases the activity dramatically, whereas the reverse effect is observed for trimethoxy derivatives. It has been shown that acetylation on geranylphenols with no methoxy groups produces a slight decrease of inhibitory activity [9]. These results confirm that the activity of geranyl compounds against the mycelial growth of *B. cinerea* is mainly determined by the geranyl chain. However, the activity of digeranyl derivatives is lower than that exhibited by the respective monogeranyl compound (comparison of **20** and **21**, **16** and **15** in Table 2). A similar behavior is observed when the activities of **5** and 2,6-digeranylphloroglucinol, a digeranyl derivative obtained from the reaction of phloroglucinol and geraniol, are compared [9]. These results are in line with previous work that established that the efficiency decreases with the increasing number of prenyl moieties [6,9].

It is worth mentioning that the effect of chemical structure on antifungal activity could be associated with differences in water solubility, which finally determines the biodisponibility of these compounds, or with their biochemical mechanism of action. This point is a very important issue that we will intend to clarify in a study where polymer micelles are used to increase the water solubility of these compounds.

## 3. Experimental Section

### 3.1. General

Unless otherwise stated, all chemical reagents purchased (Merck, Darmstadt, Germany or Aldrich, St. Louis, MO, USA) were of the highest commercially available purity and were used without previous purification. Fourier transform infrared (FT-IR) spectra were recorded as thin films in a FT-IR Nicolet 6700 spectrometer (Thermo Scientific, San Jose, CA, USA) and frequencies are reported in cm^−1^. Low resolution mass spectra were recorded on an Agilent 5973 spectrometer (Agilent Technologies, Santa Clara, CA, USA) at 70 eV ionizing voltage coupled with a 6890 N gas chromatograph equipped with a DB-5 column (30 m × 0.25 mm × 0.25 μm), and data are given as percentage of relative intensity *m*/*z* (% rel. int.). High resolution mass spectra were recorded on an LTQ Orbitrap XL spectrometer (Thermo Scientific, San Jose, CA, USA) by applying a voltage of 1.8 kV in the positive and 1.9 kV in the negative ionization mode. The spectra were recorded using full scan mode, covering a mass range from *m*/*z* 100–1300. The resolution was set to 50,000 and maximum loading time for the ion cyclotron resonance (ICR) cell was set to 250 ms. ^1^H, ^13^C, ^13^C DEPT-135, sel. gs-1D ^1^H NOESY, gs-2D HSQC and gs-2D HMBC spectra were recorded in CDCl_3_ solutions and are referenced at the residual peaks of CHCl3 at δ = 7.26 and 77.0 ppm for ^1^H and ^13^C, respectively, on a Bruker Avance 400 Digital NMR spectrometer (Bruker, Rheinstetten, Germany), operating at 400.1 MHz for ^1^H and 100.6 MHz for ^13^C. Chemical shifts are reported in δ ppm and coupling constants (*J*) are given in Hz. Silica gel (Merck 200–300 mesh) was used for C.C. and silica gel plates HF_254_ for thin layer chromatography (TLC). TLC spots were detected by heating after spraying with 25% H_2_SO_4_ in H_2_O.

### 3.2. Synthesis

#### 3.2.1. Geranylation Reaction

The coupling of geraniol and phenols were carried out using boric trifluoride etherate BF_3_·OEt_2_ as catalyst and dioxane as solvent. Alternatively, acetonitrile was used as solvent and AgNO_3_ as secondary catalyst. In a typical reaction, BF_3_·OEt_2_ (0.46 g, 3.2 mmol) is slowly added dropwise to an equimolar solution of a phenol derivative (9.1 mmol) and geraniol (1.4 g, 9.1 mmol) in dioxane (or acetonitrile saturated with AgNO_3_), with stirring at room temperature and under a N_2_ atmosphere. After the addition is completed, the stirring is continued for 48 h. The end of the reaction is verified by TLC, and then the mixture is poured onto crushed ice (30 g) and extracted with EtOAc (3 × 20 mL). The organic layer is washed with 5% NaHCO_3_ (15 mL) and water (2 × 15 mL), dried over Na_2_SO_4_, and filtered. The solvent is then evaporated under reduced pressure, and the crude is re-dissolved in CH_2_Cl_2_ (5 mL) and chromatographed on silica gel with petroleum ether/EtOAc mixtures of increasing polarity (19.8:0.2→0.2:19.8).

#### 3.2.2. Acetylation of Geranylated Phenols

Geranylated phenols were acetylated by standard acetylation using Ac_2_O and dimethylaminopyridine (DMAP) in CH_2_Cl_2_. In a typical reaction, Ac_2_O (1.08 g, 10.6 mmol) is added to a solution of a geranylated phenol (0.284 mmol), DMAP (3.0 mg) and pyridine (1.0 mL) in dichloromethane (30 mL). The end of the reaction is verified by TLC (1 h), and then the mixture is extracted with EtOAc (2 × 10 mL). The organic layer is washed with 5% KHSO_4_ (2 × 10 mL) and water (2 × 10 mL), dried over Na_2_SO_4_, and filtered. The solvent is evaporated under reduced pressure.

#### 3.2.3. Synthesis of Geranylated Methoxyphenol/Phenylacetate Derivatives

##### (*E*)-2-(3,7-Dimethylocta-2,6-dienyl)-4-methoxyphenol (**6**)

Compound **6** was obtained as colorless viscous oil (375.5 mg, 18%) by coupling of 4-methoxyphenol (1.00 g, 8.0 mmol) and geraniol (1.2 g, 7.9 mmol) in acetonitrile (15 mL), saturated with AgNO_3_, and BF_3_·OEt_2_ (0.2 g 1.6 mmol) as catalyst. NMR data of **10** was consistent with that reported in literature [15].

##### (*E*)-2-(3,7-Dimethylocta-2,6-dienyl)-4-methoxyphenyl Acetate (**7**)

Compound **7** was obtained by standard acetylation of compound **10** (90 mg, 0.346 mmol) with Ac_2_O (1.0 mL), DMAP (5.0 mg) and pyridine (2.0 mL) in dichloromethane (30 mL). Compound **7** was obtained as colorless viscous oil (99.3 mg, 95%). NMR data of compound **7** was consistent with that found in literature [15].

##### (*E*)-2-(3,7-Dimethylocta-2,6-dienyl)-6-methoxyphenyl Acetate (**9**)

Compound **9** was obtained by standard acetylation of compound **8** (50 mg, 0.192 mmol) with Ac_2_O (1.0 mL), DMAP (2.0 mg) and pyridine (1.0 mL) in dichloromethane (20 mL). Compound **13** was obtained as a pale yellow viscous oil (53.9 mg, 93%). Compound **9**: IR (cm^−1^) 2924, 1762, 1496, 1195, 1041; ^1^H NMR (CDCl_3_, 400.1 MHz) δ 7.12 (1H, dd, *J* = 8.0 and 8.0 Hz, H-4), 6.98 (1H, s, H-5), 6.82 (1H, d, *J* = 8.0 Hz, H-3), 5.23 (1H, t, *J* = 7.8 Hz, H-2ʹ), 5.09 (1H, t, *J* = 6.3 Hz, H-6ʹ), 3.81 (3H, s, CH_3_O), 3.24 (2H, d, *J* = 7.2 Hz, H-1ʹ), 2.32 (3H, s, CH_3_CO), 2.10–2.07 (2H, m, H-5ʹ), 2.04–2.03 (2H, m, H-4ʹ), 1.72 (3H, s, CH_3_-C3ʹ), 1.59 (3H, s, H-8ʹ, 1.56 (3H, s, CH_3_-C7ʹ); ^13^C NMR (CDCl_3_, 100.6 MHz) δ 168.9 (C, CH_3_CO), 151.5 (C, C-6), 145.4 (C, C-1), 135.8 (C, C-3ʹ), 131.5 (C, C-2), 128.3 (C, C-7ʹ), 126.3 (CH, C-4), 125.5 (CH, C-6ʹ), 124.2 (CH, C-2ʹ), 121.5 (CH, C-3), 109.9 (CH, C-5), 55.9 (CH_3_, CH_3_O), 39.7 (CH_2_, C-4ʹ), 29.7 (CH_2_, C-1ʹ), 28.5 (CH_2_, C-5ʹ), 25.7 (CH_3_, C-8ʹ), 20.5 (CH_3_, CH_3_CO), 17.7 (CH_3_, CH_3_-C7ʹ), 16.1 (CH_3_, CH_3_-C3ʹ).

##### (*E*)-2-(3,7-Dimethylocta-2,6-dienyl)-6-methoxyphenol (**8**), (*E*)-3-(3,7-Dimethylocta-2,6-dienyl)-2-methoxyphenol (**10**), (*E*)-5-(3,7-Dimethylocta-2,6-dien-1-yl)-2-methoxyphenol (**11**)

Guayacol (1.5 g, 12.1 mmol) and geraniol (0.9 g, 6.0 mmol) were reacted in acetonitrile (15 mL), saturated with AgNO_3_, using BF_3_·OEt_2_ (0.45 g, 3.2 mmol) as catalyst. By following the general procedure described above, three fractions were obtained: Fraction I, 70.6 mg of yellow viscous oil (4.5% yield, compound **8**); Fraction II, 98.5 mg of yellow viscous oil (6.3% yield, compound **10**); and Fraction III, 9.3 mg of yellow viscous oil (0.6% yield, compound **11**). NMR data of compounds **8**, **10** and **11** were consistent with those reported in literature [11].

##### (*E*)-2-(3,7-Dimethylocta-2,6-dienyl)-5-methoxyphenol (**12**), (*E*)-2-(3,7-Dimethylocta-2,6-dienyl)-3-methoxyphenol (**13**)

Reaction of 3-methoxyphenol (0.8 g, 6.5 mmol) and geraniol (1.0 g, 6.5 mmol) was carried out in acetonitrile (20 mL), saturated with AgNO_3_, using BF_3_·OEt_2_ (0.46 g 3.2 mmol) as catalyst. By following the general procedure described above, two fractions were obtained: Fraction I, 125 mg of reddish viscous oil (7.4% yield, compound **12**); and Fraction II, 71.1 mg of reddish viscous oil (4.2% yield, compound **13**). Compound **12**: ^1^H NMR (CDCl_3_, 400.1 MHz) δ 6.99 (1H, d, *J* = 8.0 Hz, H-3), 6.44 (1H, dd, *J* = 2.5 and 8.0 Hz, H-4), 6.42 (1H, d, *J* = 2.5 Hz, H-6), 5.31 (1H, t, *J* = 7.2 Hz, H-2ʹ), 5.25 (1H, s, OH), 5.08 (1H, t, *J* = 5.9 Hz, H-6ʹ), 3.76 (3H, s, CH_3_O), 3.31 (2H, d, *J* = 7.2 Hz, H-1ʹ), 2.13–2.12 (2H, m, H-5ʹ), 2.09–2.06 (2H, m, H-4ʹ), 4.77 (3H, s, CH_3_-C3ʹ), 1.69 (3H, s, H-8ʹ), 1.60 (3H, s, CH_3_-C7ʹ); ^13^C NMR (CDCl_3_, 100.6 MHz) δ 159.4 (C, C-5), 155.4 (C, C-1), 138.4 (C, C-3ʹ), 132.0 (C, C-7ʹ), 130.3 (CH, C-3), 123.8 (CH, C-6ʹ), 122.0 (CH, C-2ʹ), 118.9 (C, C-2), 106.1 (CH, C-4), 102.0 (CH, C-6), 55.3 (CH_3_, CH_3_O), 39.7 (CH_2_, C-4ʹ), 29.2 (CH_2_, C-1ʹ), 26.4 (CH_2_, C-5ʹ), 25.7 (CH_3_, C-8ʹ), 17.7 (CH_3_, CH_3_-C7ʹ), 16.1 (CH_3_, CH_3_-C3ʹ); HRMS *m*/*z* 261.1785 (calcd for C_17_H_24_O_2_, 261.1776). Compound **13**: ^1^H NMR (CDCl_3_, 400.1 MHz) δ 7.06 (1H, dd, *J* = 8.2 and 8.2 Hz, H-5), 6.49 (2H, d, *J* = 8.2 Hz, H-4 and H-6), 5.32 (1H, s, OH), 5.24 (1H, t, *J* = 7.0 Hz, H-2ʹ), 5.06 (1H, t, *J* = 6.6 Hz, H-6ʹ), 3.81 (3H, s, CH_3_O), 3.43 (2H, d, *J* = 7.0 Hz, H-1ʹ), 2.11–2.08 (2H, m, H-5ʹ), 2.06–2.04 (2H, m, H-4ʹ), 1.81 (3H, s, CH_3_-C3ʹ), 1.68 (3H, s, H-8ʹ), 1.59 (3H, s, CH_3_-C7ʹ); ^13^C NMR (CDCl_3_, 100.6 MHz) δ 157.9 (C, C-3), 155.6 (C, C-1), 138.1 (C, C-3ʹ), 131.8 (C, C-7ʹ), 127.1 (CH, C-5), 123.9 (CH, C-6ʹ), 121.9 (CH, C-2ʹ), 115.2 (C, C-2), 108.1 (CH, C-6), 103.1 (CH, C-4), 55.8 (CH_3_, CH_3_O), 39.7 (CH_2_, C-4ʹ), 26.4 (CH_2_, C-5ʹ), 25.6 (CH_3_, C-8ʹ), 22.2 (CH_2_, C-1ʹ), 17.7 (CH_3_, CH_3_-C7ʹ), 16.1 (CH_3_, CH_3_-C3ʹ); HRMS *m*/*z* 259.17151 (calcd for C_17_H_24_O_2_, 259.1776).

##### (*E*)-2-(3,7-Dimethylocta-2,6-dienyl)-4,6-dimethoxyphenol (**14**), 2,3-bis((*E*)-3,7-Dimethylocta-2,6-dienyl)-4,6-dimethoxyphenol (**15**), (*E*)-5-(3,7-Dimethylocta-2,6-dien-1-yl)-2,4-dimethoxyphenol (**16**)

Reaction of 2,4-dimethoxyphenol (0.807 g, 5.2 mmol) and geraniol (0.802 g, 5.2 mmol) was carried out in dioxane (20 mL) with BF_3_·OEt_2_ (0.31 g, 2.2 mmol) as catalyst. By following the general procedure described above, three fractions were obtained: Fraction I, 15.0 mg of viscous light brown oil (1.0% yield, compound **14**); Fraction II, 28.0 mg of viscous light brown oil (1.2% yield, compound **15**); Fraction III, 45.0 mg of viscous dark brown oil (3.0% yield, compound **16**). Compound **14**: IR (cm^−1^): 3556, 2964, 2919, 2854, 1613, 1497, 1466, 1432, 1376, 1227, 1197, 1148, 1089, 1055, 939, 829; ^1^H NMR (CDCl_3_, 400.1 MHz) δ 6.36 (1H, d, *J* = 2.6 Hz, H-5), 6.30 (1H, d, *J* = 2.6 Hz, H-3), 5.33 (1H, t, *J* = 7.2 Hz, H-2ʹ), 5.28 (1H, s, OH), 5.11 (1H, t, *J* = 7.7 Hz, H-6ʹ), 3.86 (3H, s, CH_3_O-C6), 3.75 (3H, s, CH_3_O-C4), 3.35 (2H, d, *J* = 7.2 Hz, H-1ʹ), 2.11–2.08 (2H, m, H-5ʹ), 2.06–2.03 (2H, m, H-4ʹ), 1.72 (3H, s, CH_3_ C-3ʹ), 1.67 (3H, s, H-8ʹ), 1.60 (3H, s, CH_3_ C-7ʹ); ^13^C NMR (CDCl_3_, 100.6 MHz) δ 152.9 (C, C-4), 146.7 (C, C-6), 137.4 (C, C-1), 136.5 (C, C-3ʹ), 131.4 (C, C-7ʹ), 127.5 (C, C-2), 124.3 (CH, C-6ʹ), 122.0 (CH, C-2ʹ), 105.5 (CH, C-3), 96.7 (CH, C-5), 56.0 (CH_3_, CH_3_O-C6), 55.7 (CH_3_, CH_3_O-C4), 39.8 (CH_2_, C-4ʹ), 28.1 (CH_2_, C-1ʹ), 26.7 (CH_2_, C-5ʹ), 25.7 (CH_3_, C-8ʹ), 17.7 (CH_3_, CH_3_-C7ʹ), 16.1 (CH_3_, CH_3_-C3ʹ); MS *m*/*z* 290 (M^+^) (100), 205 (24), 189 (19), 168 (65) ((M^+^ + H − 123), (C_9_H_15_·)), 167 (39), 166 (45), 161 (29), 69 (18), 41 (21). NMR data of compound **14** was consistent with that in the literature [6]. Compound **15**: IR (cm^−1^): 3556, 2965, 2925, 2854, 1616, 1485, 1451, 1437, 1376, 1341, 1237, 1202, 1089, 941, 855; ^1^H NMR (CDCl_3_, 400.1 MHz) δ 6.42 (1H, s, H-5), 5.31 (1H, s, OH), 5.12–5.06 (4H, m, H-2ʹ, H-2ʹʹ, H-6ʹ and H-6ʹʹ), 3.87 (3H, s, CH_3_O-C6), 3.77 (3H, s, CH_3_O-C4), 3.39 (2H, d, *J* = 6.0 Hz, H-1ʹ), 3.31 (2H, d, *J* = 6.0 Hz, H-1ʹʹ), 2.07–2.05 (4H, m, H-5ʹ and H-5ʹʹ), 2.03–1.98 (4H, m, H-4ʹ and H-4ʹʹ), 1.76 (3H, s, CH_3_-C3ʹ), 1.74 (3H, s, CH_3_-C3ʹ), 1.66 (6H, s, H-8ʹ and H-8ʹʹ), 1.58 (6H, s, CH_3_-C7ʹ and CH_3_-C7ʹʹ); ^13^C NMR (CDCl_3_, 100.6 MHz) δ 150.7 (C, C-4), 144.4 (C, C-6), 137.9 (C, C-1), 135.2 (C, C-3ʹ), 134.4 (C, C-3ʹʹ), 131.2 (C, C-7ʹʹ and C-7ʹ), 127.3 (C, C-2), 124.4 (CH, C-6ʹ and C-6ʹʹ), 123.8 (CH, C-2ʹ), 122.8 (CH, C-2ʹʹ), 122.1 (C, C-3), 95.3 (C, C-5), 56.9 (CH_3_, CH_3_O-C4), 56.1 (CH_3_, CH_3_O-C6), 39.7 (CH_2_, C-4ʹ and C-4ʹʹ), 26.7 (CH_2_, C-5ʹ and C-5ʹʹ), 25.6 (CH_3_, C-8ʹ and C-8ʹʹ), 25.3 (CH_2_, C-1ʹʹ), 24.6 (CH_2_, C-1ʹ), 17.6 (CH_3_, CH_3_-C7ʹ and CH_3_-C7ʹʹ), 16.2 (CH_3_, CH_3_-C3ʹ), 16.1 (CH_3_, CH_3_-C3ʹʹ); MS *m*/*z* 426 (M^+^) (74), 302 (16), 259 (32), 220 (40), 219 (100), 201 (41), 187 (63), 167 (19), 69 (67), 41 (48); HRMS *m*/*z* 427.3141 (calcd for C_28_H_42_O_3_, 427.3134). Compound **16**: IR (cm^−1^) 2924, 1602, 1512, 1194, 1042; ^1^H NMR (CDCl_3_, 400.1 MHz) δ 6.68 (1H, s, H-6), 6.54 (1H, s, H-3), 5.51 (1H, s, OH), 5.28 (1H, t, *J* = 7.1 Hz, H-2ʹ), 5.11 (1H, t, *J* = 6.9 Hz, H-6ʹ), 3.82 (3H, s, CH_3_O-C2), 3.76 (3H, s, CH_3_O-C4), 3.26 (2H, d, *J* = 7.2 Hz, H-1ʹ), 2.12–2.08 (2H, m, H-5ʹ), 2.06–2.02 (2H, m, H-4ʹ), 1.70 (3H, s, CH_3_-C3ʹ), 1.67 (3H, s, H-8ʹ), 1.60 (3H, s, CH_3_-C7ʹ); ^13^C NMR (CDCl_3_, 100.6 MHz) δ 151.7 (C, C-4), 144.1 (C, C-1), 140.1 (C, C-2), 136.0 (C, C-3ʹ), 131.4 (C, C-7ʹ), 124.3 (CH, C-6ʹ), 122.8 (CH, C-2ʹ), 120.9 (C, C-5), 112.8 (CH, C-6), 99.3 (CH, C-3), 56.8 (CH_3_, CH_3_O-C2), 56.1 (CH_3_, CH_3_O-C4), 39.8 (CH_2_, C-4ʹ), 27.7 (CH_2_, C-1ʹ), 26.8 (CH_2_, C-5ʹ), 25.7 (CH_3_, C-8ʹ), 17.7 (CH_3_, CH_3_-C7ʹ), 16.1 (CH_3_, CH_3_-C3ʹ); MS *m*/*z* 290 (M^+^) (100), 221 (15), 207 (12), 189 (39), 178 (22), 167 (84) ((M^+^ − 123), (C_9_H_15_·)), 161 (68), 129 (26), 69 (24), 41 (27).

##### (*E*)-6-(3,7-Dimethylocta-2,6-dienyl)-2,3,4-trimethoxyphenol (**17**) and (*E*)-5-(3,7-Dimethylocta-2,6-dien-1-yl)-2,3,4-trimethoxyphenol (**18**)

Coupling of 2,3,4-trimethoxyphenol (1.0 g, 5.5 mmol) and geraniol (0.85 g, 5.5 mmol) was carried out in dioxane (20 mL) with BF_3_·OEt_2_ (0.46 g, 3.2 mmol) as catalyst. By following the general procedure described above, two fractions were obtained: Fraction I, 150.5 mg of viscous light brown oil (8.6% yield, compound **17**); Fraction II, 71.0 mg of viscous dark brown oil (4.1% yield, compound **18**). Compound **17**: IR (cm^−1^) 3446; 2966; 1497; 1464; 1125; 1072; ^1^H NMR (CDCl_3_, 400.1 MHz) δ 6.44 (1H, s, H-5), 5.47 (1H, s, OH), 5.32 (1H, t, *J* = 6.8 Hz, H-2ʹ), 5.11 (1H, t, *J* = 6.6 Hz, H-6ʹ), 3.95 (3H, s, CH_3_O-C2), 3.87 (3H, s, CH_3_O-C3), 3.79 (3H, s, CH_3_O-C4), 3.31 (2H, d, *J* = 7.2 Hz, H-1ʹ), 2.11–2.09 (2H, m, H-5ʹ), 2.07–2.04 (2H, m, H-4ʹ), 1.72 (3H, s, CH_3_ C-3ʹ), 1.67 (3H, s, H-8ʹ), 1.60 (3H, s, CH_3_ C-7ʹ); ^13^C NMR (CDCl_3_, 100.6 MHz) δ 146.1 (C, C-4), 140.8 (C, C-1), 140.0 (C, C-2 and C-3), 136.6 (C, C-3ʹ), 131.4 (C, C-7ʹ), 124.2 (CH, C-6ʹ), 122.0 (CH, C-2ʹ), 121.6 (CH, C-6), 108.2 (C, C-5), 61.2 (CH_3_, CH_3_O-C2), 60.9 (CH_3_, CH_3_O-C3), 56.6 (CH_3_, CH_3_O-C4), 39.7 (CH_2_, C-4ʹ), 27.9 (CH_2_, C-1ʹ), 26.7 (CH_2_, C-5ʹ), 25.7 (CH_3_, C-8ʹ), 17.7 (CH_3_, CH_3_-C7ʹ), 16.1 (CH_3_, CH_3_-C3ʹ); MS *m*/*z* 320 (M^+^) (71), 235 (15), 198 (18), 197 (100) ((M^+^ − 123), (C_9_H_15_·)), 181 (12), 159 (12), 69 (9), 41 (12). NMR data of compound **17** was consistent with that reported in the literature [6].

Compound **18**: IR (cm^−1^) 3421, 2966, 1590, 1487, 1465, 1198, 1110; ^1^H NMR (CDCl_3_, 400.1 MHz) δ 6.50 (1H, s, H-6), 5.46 (1H, s, OH), 5.25 (1H, t, *J* = 6.2 Hz, H-2ʹ),5.10 (1H, bt, *J* = 6.8 Hz, H-6ʹ), 3.93 (3H, s, CH_3_O-C3), 3.92 (3H, s, CH_3_O-C2), 3.78 (3H, s, CH_3_O-C4), 3.27 (2H, d, *J* = 7.2 Hz, H-1ʹ), 2.11–2.08 (2H, m, H-5ʹ), 2.05–2.02 (2H, m, H-4ʹ), 1.70 (3H, s, CH_3_-C3ʹ), 1.68 (3H, s, H-8ʹ), 1.60 (3H, s, CH_3_-C7ʹ); ^13^C NMR (CDCl_3_, 100.6 MHz) δ 145.9 (C, C-3), 144.9 (C, C-4), 144.5 (C, C-1), 138.0 (C, C-2), 136.2 (C, C-3ʹ), 131.5 (C, C-7ʹ), 130.6 (C, C-5), 124.2 (CH, C-6ʹ), 122.5 (CH, C-2ʹ), 109.5 (CH, C-6), 61.2 (CH_3_, CH_3_O-C3), 61.0 (CH_3_, CH_3_O-C2), 60.7 (CH_3_, CH_3_O-C4), 39.7 (CH_2_, C-4ʹ), 27.8 (CH_2_, C-1ʹ), 26.6 (CH_2_, C-5ʹ), 25.7 (CH_3_, C-8ʹ), 17.7 (CH_3_, CH_3_-C7ʹ), 16.0 (CH_3_, CH_3_-C3ʹ); MS *m*/*z* 320 (M^+^) (100), 219 (33), 206 (26), 197 (75) ((M^+^ − 123), (C_9_H_15_·)), 183 (18), 159 (49), 69 (25), 41 (27); HRMS *m*/*z* 321.1994 (calcd for C_19_H_28_O_4_, 321.1988).

##### (*E*)-6-(3,7-Dimethylocta-2,6-dienyl)-2,3,4-trimethoxyphenyl Acetate (**19**)

Standard acetylation of compound **17** (48 mg, 0.150 mmol) with Ac_2_O (1.08 g, 10.6 mmol), DMAP (3.0 mg) and pyridine (1.0 mL) in dichloromethane (20 mL) gives compound **19** as a viscous light brown oil (54 mg, 99.4% yield). Compound **19**: IR (cm^−1^) 2935, 1766, 1587, 1492, 1463, 1124, 1071; ^1^H NMR (CDCl_3_, 400.1 MHz) δ 6.49 (1H, s, H-5), 5.22 (1H, t, *J* = 7.0 Hz, H-2ʹ), 5.10 (1H, t, *J* = 6.4 Hz, H-6ʹ), 3.87 (3H, s, CH_3_O-C3), 3.86 (3H, s, CH_3_O-C2), 3.83 (3H, s, CH_3_O-C4), 3.18 (2H, d, *J* = 7.2 Hz, H-1ʹ), 2.31 (3H, s, CH_3_CO), 2.11–2.07 (2H, m, H-5ʹ), 2.05–2.01 (2H, m, H-4ʹ), 1.69 (3H, s, CH_3_-C3ʹ), 1.67 (3H, s, H-8ʹ), 1.61 (3H, s, CH_3_-C7ʹ); ^13^C NMR (CDCl_3_, 100.6 MHz) δ 169.4 (C, COCH_3_), 151.2 (C, C-4), 145.6 (C, C-2), 140.9 (C, C-3), 136.9 (C, C-1), 135.8 (C, C-6), 131.5 (C, C-7ʹ), 128.9 (C, C-3ʹ), 124.1 (CH, C-6ʹ), 121.4 (CH, C-2ʹ), 107.1 (CH, C-5), 61.0 (CH_3_, CH_3_O-C2), 60.9 (CH_3_, CH_3_O-C3), 56.1 (CH_3_, CH_3_O-C4), 39.7 (CH_2_, C-4ʹ), 28.6 (CH_2_, C-1ʹ), 26.7 (CH_2_, C-5ʹ), 25.7 (CH_3_, C-8ʹ), 20.4 (CH_3_, COCH_3_), 17.7 (CH_3_, CH_3_-C7ʹ), 16.2 (CH_3_, CH_3_-C3ʹ); MS *m*/*z* 362 (M^+^) (11), 320 (32), 235 (16), 221 (10), 197 (100) ((M^+^ − 123), (C_9_H_15_·)), 181 (9), 159 (10), 69 (10), 41 (13).

##### (*E*)-3-(3,7-Dimethylocta-2,6-dien-1-yl)-2,4,6-trimethoxyphenol (**20**) and 3,5-bis((*E*)-3,7-Dimethylocta-2,6-dien-1-yl)-2,4,6-trimethoxyphenol (**21**)

Coupling of 2,4,6-trimethoxyphenol (2.58 g, 14.0 mmol) and geraniol (2.49 g, 16.0 mmol) was carried out in dioxane (20 mL) with BF_3_·OEt_2_ (0.90 g, 6.3 mmol) as catalyst. Two fractions were obtained: Fraction I, 735.9 mg of viscous brown oil (16.4% yield, compound **20**); Fraction II, 901.8 mg of viscous light brown oil (14.0% yield, compound **21**). Compound **20**: IR (cm^−1^) 3447, 2965, 2929, 2838, 1668, 1615, 1500, 1454, 1377, 1345, 1245, 1199, 1105, 910, 872, 801; ^1^H NMR (CDCl_3_, 400.1 MHz) δ 6.32 (1H, s, H-5), 5.17 (1H, t, *J* = 6.2 Hz, H-2ʹ), 5.15 (1H, s, OH), 5.06 (1H, t, *J* = 6.7 Hz, H-6ʹ), 3.88 (3H, s, CH_3_O-C6), 3.83 (3H, s, CH_3_O-C2), 3.78 (3H, s, CH_3_O-C4), 3.30 (2H, d, *J* = 6.8 Hz, H-1ʹ), 2.06–2.02 (2H, m, H-5ʹ), 1.98–1.94 (2H, m, H-4ʹ), 1.76 (3H, s, CH_3_-C3ʹ), 1.64 (3H, s, H-8ʹ), 1.57 (3H, s, CH_3_-C7ʹ); ^13^C NMR (CDCl_3_, 100.6 MHz) δ 150.6 (C, C-4), 145.9 (C, C-2), 145.3 (C, C-6), 134.4 (C, C-3ʹ), 133.0 (C, C-1), 131.1 (C, C-7ʹ), 124.4 (CH, C-6ʹ), 123.5 (CH, C-2ʹ), 116.8 (C, C-3), 93.0 (CH, C-5), 60.8 (CH_3_, CH_3_O-C2), 56.5 (CH_3_, CH_3_O-C4), 56.3 (CH_3_, CH_3_O-C6), 39.7 (CH_2_, C-4ʹ), 26.7 (CH_2_, C-5ʹ), 25.6 (CH_3_, C-8ʹ), 22.5 (CH_2_, C-1ʹ), 17.6 (CH_3_, CH_3_-C7ʹ), 16.0 (CH_3_, CH_3_-C3ʹ); MS *m*/*z* 320 (M^+^) (77), 251 (22), 237 (16), 221 (31), 219 (33), 197 (100) ((M^+^ − 123), (C_9_H_15_·)), 186 (27), 183 (24), 159 (17), 69 (18), 41 (23). Compound **21**: IR (cm^−1^) 3447, 3086, 2968, 2926, 2856, 1675, 1642, 1604, 1460, 1422, 1376, 1235, 1106, 1031, 920, 835; ^1^H NMR (CDCl_3_, 400.1 MHz) δ 5.34 (1H, s, OH), 5.24–5.20 (2H, m, H-2ʹ and H-2ʹʹ), 5.08 (2H, m, H-6ʹ and H-6ʹʹ), 3.81 (6H, s, CH_3_O-C6 and CH_3_O-C2), 3.66 (3H, s, CH_3_O-C4), 3.34 (4H, d, *J* = 6.6 Hz, H-1ʹ and H-1ʹʹ), 2.07–2.04 (4H, m, H-5ʹ and H-5ʹʹ), 2.01–1.97 (4H, m, H-4ʹ and H-4ʹʹ), 1.77 (6H, s, CH_3_-C3ʹ and CH_3_-C3ʹʹ), 1.64 (6H, s, H-8ʹ and H-8ʹʹ), 1.57 (6H, s, CH_3_-C7ʹ and CH_3_-C7ʹʹ); ^13^C NMR (CDCl_3_, 100.6 MHz) δ 149.5 (C, C-4), 144.4 (C, C-2 and C-6), 139.0 (C, C-1), 134.8 (C, C-3ʹ and C-3ʹʹ), 131.3 (C, C-7ʹ and C-7ʹʹ), 124.4 (C, C-3 and C-5), 124.3 (C, C-6ʹ and C-6ʹʹ), 123.8 (CH, C-2ʹ and C-2ʹʹ), 61.7 (CH_3_, CH_3_O-C4), 60.9 (CH_3_, CH_3_O-C2 and CH_3_O-C6), 39.7 (CH_2_, C-4ʹ and C-4ʹʹ), 26.6 (CH_2_, C-5ʹ and C-5ʹʹ), 25.6 (CH_3_, C-8ʹ and C-8ʹʹ), 23.5 (CH_2_, C-1ʹ and C-1ʹʹ), 17.6 (CH_3_, CH_3_-C7ʹ and CH_3_-C7ʹʹ), 16.2 (CH_3_, CH_3_-C3ʹ and CH_3_-C3ʹʹ); MS *m*/*z* 456 (M^+^) (33), 333 (22) ((M^+^ − 123), (C_9_H_15_·)), 301 (11), 263 (100), 211 (34), 69 (54), 41 (38); HRMS *m*/*z* 457.3247 (calcd for C_29_H_44_O_4_, 457.3240).

##### (*E*)-3-(3,7-Dimethylocta-2,6-dienyl)-2,4,6-trimethoxyphenyl Acetate (**22**)

Standard acetylation of compound **20** (65 mg, 0.18 mmol) with Ac_2_O (0.5 mL, 5.3 mmol), DMAP (5.0 mg) and pyridine (1.0 mL) in dichloromethane (30 mL) gives compound **22** as a viscous yellow oil (72 mg, 99% yield). Compound **22**: IR (cm^−1^) 3402, 2961, 2970, 2843, 1767, 1607, 1496, 1458, 1375, 1201, 1108, 1024, 927; ^1^H NMR (CDCl_3_, 400.1 MHz) δ 6.32 (1H, s, H-5), 5.16 (1H, t, *J* = 6.4 Hz, H-2ʹ), 5.06 (1H, t, *J* = 6.8 Hz, H-6ʹ), 3.81 (6H, s, CH_3_O-C4 and CH_3_O-C6), 3.74 (3H, s, CH_3_O-C2), 3.29 (2H, d, *J* = 6.7 Hz, H-1ʹ), 2.33 (3H, s, COCH_3_), 2.05–2.02 (2H, m, H-5ʹ), 1.98–1.94 (2H, m, H-4ʹ), 1.75 (3H, s, CH_3_-C3ʹ), 1.64 (3H, s, H-8ʹ), 1.57 (3H, s, CH_3_-C7ʹ); ^13^C NMR (CDCl_3_, 100.6 MHz) δ 169.1 (C, COCH_3_), 155.8 (C, C-4), 151.3 (C, C-2), 150.2 (C, C-6), 134.5 (C, C-3ʹ), 131.1 (C, C-7ʹ), 127.0 (C, C-1), 124.4 (CH, C-6ʹ), 123.2 (CH, C-2ʹ), 116.4 (C, C-3), 92.5 (CH, C-5), 61.3 (CH_3_, CH_3_O-C2), 56.1 (CH_3_, CH_3_O-C4), 55.8 (CH_3_, CH_3_O-C6), 39.7 (CH_2_, C-4ʹ), 26.6 (CH_2_, C-5ʹ), 25.6 (CH_3_, C-8ʹ), 22.5 (CH_2_, C-1ʹ), 20.4 (CH_3_, COCH_3_), 17.6 (CH_3_, CH_3_-C7ʹ), 15.9 (CH_3_, CH_3_-C3ʹ).

##### (*E*)-5-(3,7-Dimethylocta-2,6-dienyl)-2,4-dimethoxyphenol (**16**), (*E*)-2-(3,7-Dimethylocta-2,6-dienyl)-4,5-dimethoxyphenol (**23**)

Reaction of 2,4,5-trimethoxyphenol (2.46 g, 13.3 mmol) and geraniol (2.07 g, 13.3 mmol) was carried out in dioxane (20 mL) with BF3·OEt2 (0.62g, 4.4 mmol) as catalyst. By following the described general procedure of geranylation reaction, two fractions were obtained: Fraction I, 719.6 mg of viscous brown oil (18.7% yield, compound **23**); Fraction II, 677.5 mg of viscous light brown oil (17.3% yield, compound **16**). Spectroscopic data of compound **16** was consistent with those found above. Compound **23**: IR (cm^−1^) 2924, 1619, 1512, 1451, 1195; ^1^H NMR (CDCl_3_, 400.1 MHz) δ 6.62 (1H, s, H-3), 6.45 (1H, s, H-6), 5.30 (1H, t, *J* = 7.1 Hz, H-2ʹ), 5.07 (1H, t, *J* = 7.1 Hz, H-6ʹ), 4.93 (1H, s, OH), 3.82 (6H, s, OCH_3_ × 2), 3.30 (2H, d, *J* = 7.0 Hz, H-1ʹ), 2.09 (4H, m, H-5ʹ and H-4ʹ), 1.78 (3H, s, CH_3_-C3ʹ), 1.68 (3H, s, H-8ʹ), 1.60 (3H, s, CH_3_-C7ʹ); ^13^C NMR (CDCl_3_, 100.6 MHz) δ 148.4 (C, C-1), 148.3 (C, C-5), 142.8 (C, C-4), 138.6 (C, C-3ʹ), 132.0 (C, C-7ʹ), 123.7 (CH, C-6ʹ), 121.9 (CH, C-2ʹ), 117.3 (C, C-2), 113.7 (CH, C-3), 101.2 (CH, C-6), 56.6 (CH_3_, CH_3_O-C5), 55.9 (CH_3_, CH_3_O-C4), 39.6 (CH_2_, C-4ʹ), 29.6 (CH_2_, C-1ʹ), 26.4 (CH_2_, C-5ʹ), 25.6 (CH_3_, C-8ʹ), 17.7 (CH_3_, CH_3_-C7ʹ), 16.2 (CH_3_, CH_3_-C3ʹ); MS *m*/*z* 290 (M^+^) (45), 205 (19), 167 (100) ((M^+^ − 123), (C_9_H_15_·)), 69 (11), 41 (13). NMR data of compound **27** was consistent with those in the literature [6,10].

##### (*E*)-2-(3,7-Dimethylocta-2,6-dienyl)-4,5-dimethoxyphenyl Acetate (**24**)

Standard acetylation of compound **23** (50.8 mg, 0.175 mmol) with Ac_2_O (100 µL), DMAP (3.0 mg) and pyridine (1.0 mL) in dichloromethane (20 mL) gives compound **24** as a dark brown oil (57.2 mg, 98.3% yield). Compound **24**: IR (cm^−1^) 2973, 2929, 2854, 1765, 1616, 1514, 1448, 1369, 1206, 1101, 1015, 907, 855; ^1^H NMR (CDCl_3_, 400.1 MHz) δ 6.70 (1H, s, H-3), 6.56 (1H, s, H-6), 5.23 (1H, t, *J* = 6.9 Hz, H-2ʹ), 5.10 (1H, t, *J* = 6.4 Hz, H-6ʹ), 3.84 (3H, s, CH_3_O-C4), 3.83 (3H, s, CH_3_O-C5), 3.17 (2H, d, *J* = 7.0 Hz, H-1ʹ), 2.29 (3H, s, COCH_3_), 2.09–2.05 (2H, m, H-5ʹ), 2.05–2.04 (2H, m, H-4ʹ), 1.69 (3H, s, CH_3_-C3ʹ), 1.67 (3H, s, H-8ʹ), 1.60 (3H, s, CH_3_-C7ʹ); ^13^C NMR (CDCl_3_, 100.6 MHz) δ 169.8 (C, COCH_3_), 147.5 (C, C-5), 146.9 (C, C-4), 141.8 (C, C-1), 136.8 (C, C-3ʹ), 131.5 (C, C-7ʹ), 124.8 (C, C-2), 124.1 (CH, C-6ʹ), 121.8 (CH, C-2ʹ), 112.3 (CH, C-3), 106.1 (CH, C-6), 56.1 (CH_3_, CH_3_O-C4), 56.0 (CH_3_, CH_3_O-C5), 39.7 (CH_2_, C-4ʹ), 28.2 (CH_2_, C-1ʹ), 26.7 (CH_2_, C-5ʹ), 25.7 (CH_3_, C-8ʹ), 20.8 (CH_3_, COCH_3_), 17.7 (CH_3_, CH_3_-C7ʹ), 16.2 (CH_3_, CH_3_-C3ʹ); MS *m*/*z* 332 (M^+^) (39), 290 (34), 221 (16), 205 (25), 167 (100) ((M^+^ − CH_3_CO − 123), (C_9_H_15_·)), 69 (13), 41 (13).

#### 3.2.4. Synthesis of Trimethoxyphenols

In a typical reaction, *m*-chloroperoxybenzoic acid (mCPBA) is added to a stirred solution of trimethoxybenzaldehyde and sodium bicarbonate in dichloromethane (70 mL). The mixture is stirred at room temperature until the end of the reaction is reached (3 h). Then, the reaction mixture is filtered in a vacuum; the organic phase is washed with NaHCO_3_ (3 × 50 mL) and water (2 × 50 mL), and dried over anhydrous Na_2_SO_4_. The solvent is evaporated under reduced pressure, whereas the crude is re-dissolved in CH2Cl2 (5 mL) and chromatographed on silica gel with petroleum ether/EtOAc of increasing polarity mixtures (50:0→30:20). Trimethoxyphenyl formate is obtained as a dark brown solid. Then, triethylamine (2 mL) is added to a solution of trimethoxyphenyl formate in methanol (50 mL), and the mixture is stirred at room temperature until the end of reaction is verified by TLC (3 h). The solvent is evaporated under reduced pressure; the crude is diluted with ethyl acetate (20 mL) and the organic phase is washed with 5% HCl (2 × 30 mL) and water (2 × 15 mL), and dried over anhydrous Na_2_SO_4_. The solvent is evaporated under reduced pressure; the crude is re-dissolved in CH2Cl2 (5 mL) and chromatographed on silica gel with petroleum ether/EtOAc of increasing polarity mixtures (50:0→30:20).

2,3,4-Trimethoxyphenol. mCPBA (3.30 g, 19 mmol) was added to a solution of 2,3,4-trimethoxybenzaldehyde (2.0 g, 10 mmol) and sodium bicarbonate (1.82 g, 21 mmol) in dichloromethane (70 mL). 2,3,4-trimethoxyphenyl formate was obtained as a dark brown solid (1.81 g, 89.2% yield). Triethylamine (2 mL; 1.45 g; 14.0 mmol) was added to a solution of 2,3,4-trimethoxyphenyl formate (1.7 g, 8.0 mmol) in methanol (50 mL). 2,3,4-trimethoxyphenol was obtained as a dark brown oil (1.80 g, 99.6% yield). IR (cm^−1^) 3421, 2995, 2940, 1601, 1479, 1427, 1360, 1052, 955, 797; ^1^H NMR (CDCl_3_, 400.1 MHz) δ 6.62 (1H, d, *J* = 9.0 Hz, H-6), 6.55 (1H, d, *J* = 9.0 Hz, H-5), 3.95 (3H, s, CH_3_O), 3.89 (3H, s, CH_3_O), 3.80 (3H, s, CH_3_O); ^13^C NMR (CDCl_3_, 100.6 MHz) δ 146.9 (C, C-1), 143.3 (C, C-2), 142.2 (C, C-3), 140.4(C, C-4), 108.5 (CH, C-6), 107.6 (CH, C-5), 61.2 (CH_3_, CH_3_O), 60.9 (CH_3_, CH_3_O), 55.5 (CH3, CH_3_O).

2,4,6*-*Trimethoxyphenol. mCPBA (4.45 g, 25.8 mmol) was added to a solution of 2,4,6-trimethoxybenzaldehyde (3.03 g, 15.4 mmol) and sodium bicarbonate (2.70 g, 32.1 mmol) in dichloromethane (70 mL). 2,4,6-trimethoxyphenyl formate was obtained as a dark brown solid (2.95 g, 90.2% yield). Triethylamine (6 mL, 42.0 mmol) was added to a solution of 2,4,6-trimethoxyphenyl formate (2.95 g, 13.9 mmol) in methanol (75 mL). 2,4,6-trimethoxyphenol was obtained as a dark brown solid (2.44 g, 95.0% yield). mp 60.5–62.9 °C; IR (cm^−1^) 3422, 3010, 2964, 1626, 1469, 1439, 1361, 1236, 1053, 942, 815; ^1^H NMR (CDCl_3_, 400.1 MHz) δ 6.16 (2H, s, H-3 and H-5), 3.83 (6H, s, 2 × CH_3_O), 3.74 (3H, s, CH_3_O); ^13^C NMR (CDCl_3_, 100.6 MHz) δ 153.0 (C, C-1), 147.2 (C, C-2 and C-6), 128.9 (C, C-4), 91.7 (CH, C-3 and C-5), 56.1 (CH_3_, 2 × CH_3_O), 55.6 (CH_3_, CH_3_O).

2,4,5-Trimethoxyphenol. mCPBA (5.0 g, 29.0 mmol) was added to a solution of 2,4,5-trimethoxybenzaldehyde (3.01 g, 15.0 mmol) and sodium bicarbonate (1.8 g, 32.0 mmol) in dichloromethane (70 mL). 2,4,5-trimethoxyphenyl formate was obtained as a dark brown solid (2.99 g, 91.6% yield). Triethylamine (4 mL, 28.0 mmol) was added to a solution of 2,4,5-trimethoxyphenyl formate (2.99 g, 14.0 mmol) in methanol (75 mL). 2,4,5-trimethoxyphenol was obtained as a dark brown solid (2.46 g, 94.3% yield). mp 83.5–87.0 °C; IR (cm^−1^) 3480, 3067, 2996, 1621, 1479, 1441, 1375, 1209, 1027, 823M; ^1^H NMR (CDCl_3_, 400.1 MHz) δ 6.61 (1H, s, H-6), 6.57 (1H, s, H-3), 5.29 (1H, s, OH), 3.87 (3H, s, CH_3_O), 3.84 (6H, s, 2 × CH_3_O); ^13^C NMR (CDCl_3_, 100.6 MHz) δ 144.2 (C, C-2), 143.6 (C, C-5), 143.1 (C, C-1), 141.9 (C, C-4), 104.8 (CH, C-3), 100.1 (CH, C-6).

### 3.3. Fungal Isolate and Culture Condition

In this study, the strain UK of *B. cinerea* was used in all experiments. This strain was isolated from a naturally infected grape (*Vitis vinifera*) and was maintained on potato dextrose agar medium (PDA; Difco, Detroit, MI, USA) at 4 °C. The inoculum of the pathogen was grown on PDA in photoperiod of 16 h light/8 h dark at 23 °C for five days.

### 3.4. Effect of the Compounds on the Mycelial Growth of B. cinerea in Vitro

The anti-phytophatogenic activities of compounds, the negative control (C−), and the positive control (C+, commercial fungicide Captan) were assessed using the radial growth test technique on PDA medium [18]. All tested compounds were dissolved in ethanol (1%) and water, added at different amounts to obtain final concentrations of 50, 150 and 250 mg/L in PDA medium. Negative and positive control experimental conditions for the growth of mycelia of *B. cinerea* were included. Negative control conditions means PDA medium containing 1% ethanol, whereas positive control indicates PDA medium including the commercial fungicide Captan at the same concentration specified for the compounds of interest.

A plug (4 mm) of PDA medium with five-day-old mycelium colonies of the pathogen was placed at the center of a Petri dish with PDA medium with or without studied compounds. Subsequently, they were incubated under controlled conditions of temperature to 23 °C and photoperiod 16 h light/8 h for 48 h. The percentage of inhibition was determined for each compound by expressing the area of *B. cinerea* as a percentage of the negative control. The evaluation was conducted through measuring diameters of mycelial growth after 72 h of incubation. The inhibition percentages of mycelial growth were calculated for each compound and compared with the negative control according to Hou *et al.* [19]. All treatments were performed independently three times in triplicate.

## 4. Conclusions

The evaluation of the inhibitory effect of a series of geranylphenols on the mycelial growth of the plant pathogen *B. cinerea* was made *in vitro* after 72 h of incubation using the radial growth test technique on PDA medium, and included a commercial fungicide as a positive control. The results show that most of the compounds exhibit activities against the mycelial growth of *B. cinerea* in the range of 51% and 98% between 150 and 250 ppm, whereas compounds **6**, **8**, **10**, **12**, **15** and **22** are less active with a percentage of inhibition lower than 50%. The analysis of the relation between the activity and the structure of compounds indicates that the biological activity depends strongly on the number of methoxy, hydroxyl, acetate, and prenyl groups. For geranylphenols, the activity improves from 40% to 90% by increasing the number of methoxy groups in the aromatic ring. The position of the methoxy relative to the hydroxyl group has also a slight effect on the activity. On the other hand, for geranylacetates, the opposite effect is observed, *i.e.*, the activity decreases with an increasing number of methoxy groups. Finally, the activity of digeranyl derivatives is lower than that exhibited by the respective monogeranyl compounds. These results confirm that the activity of geranyl compounds against the mycelial growth of *B. cinerea* is mainly determined by the geranyl chain.

These structural effects may be related to differences in the response of the plant pathogen by activating or inhibiting different mechanisms (collapse of the hyphae, degradation of fungus cellular wall, or loss of turgidity) which may lead to growth arrest of the pathogenic hyphae. An alternative explanation could be the different biodisponibility of these compounds due to differences in water solubility. Thus, additional studies are required to elucidate what is the reason for the observed effects of these compounds that affect the fungus growth.

## Supplementary Materials

Supplementary materials can be found at http://www.mdpi.com/1422-0067/16/08/19130/s1.
