# Supplementary material for: Synthesis and in Vitro Antifungal Activity against Botrytis cinerea of Geranylated Phenols and Their Phenyl Acetate Derivatives
Source: Int J Mol Sci. 2015 Aug 14;16(8):19130–52. doi: 10.3390/ijms160819130 (PMC4581290; doi:10.3390/ijms160819130)
Supplement: Supplementary file 1 [file ijms-16-19130-s001.pdf]

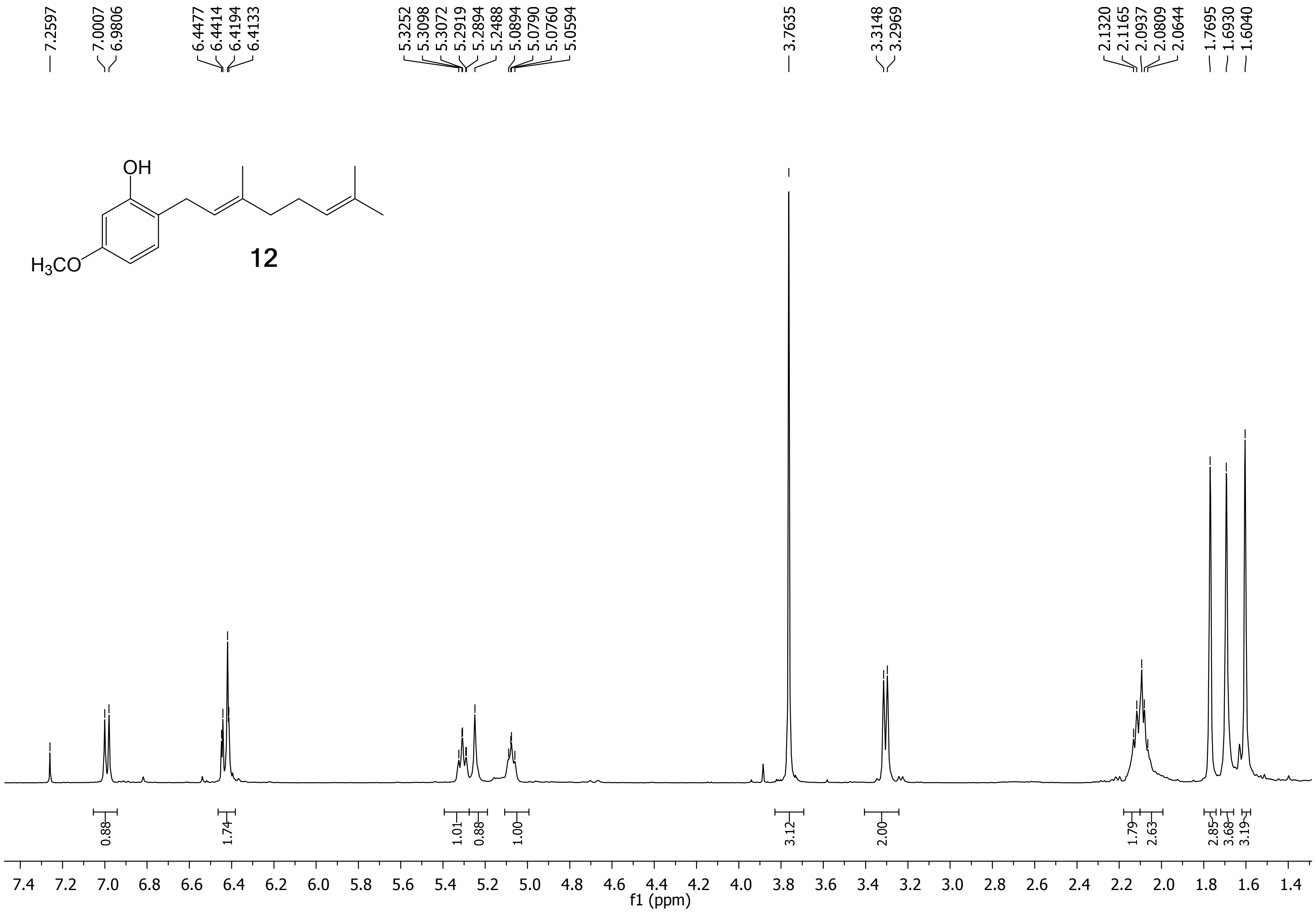

Figure S1. *Cont.*

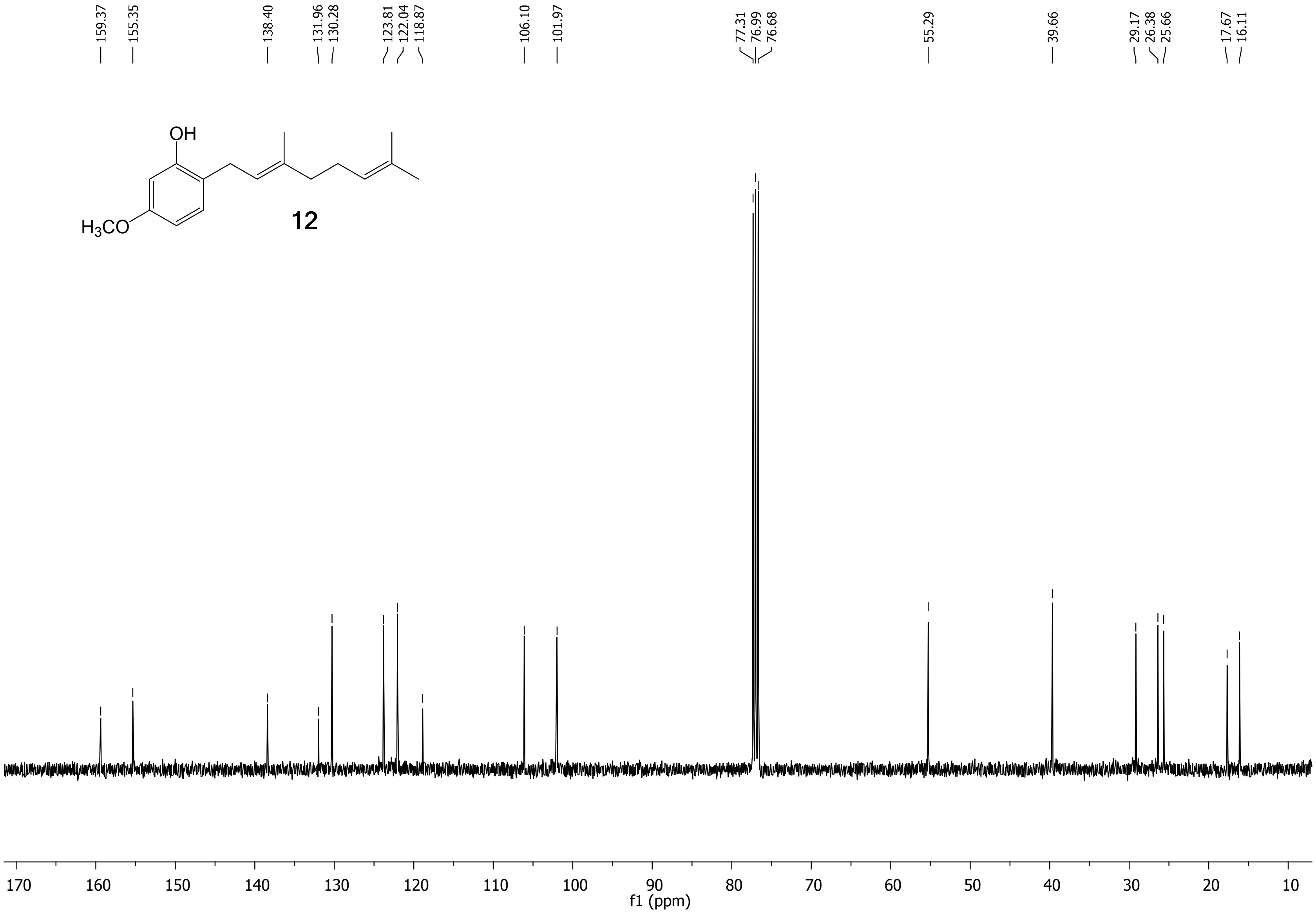

Figure S1. *Cont.*

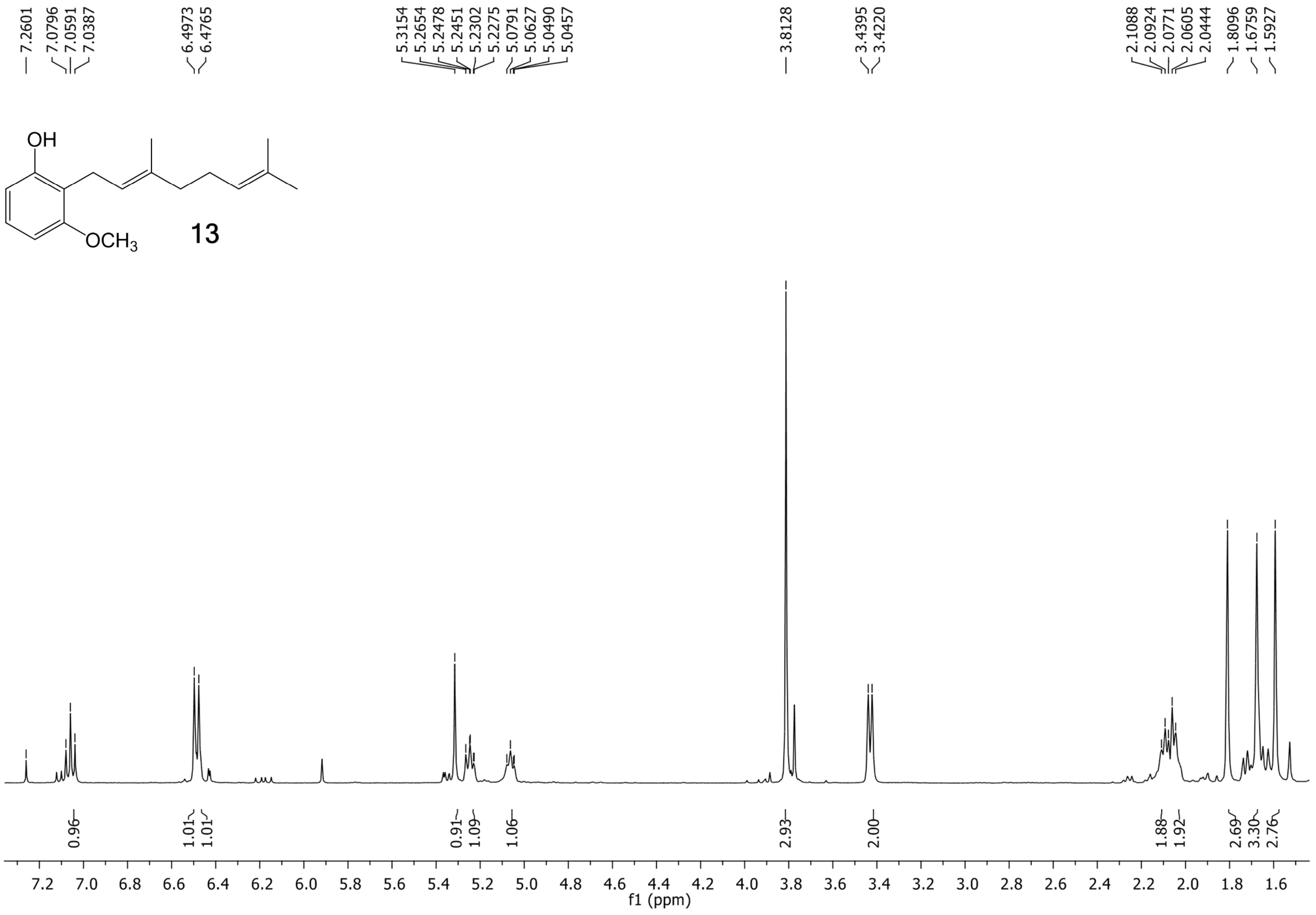

Figure S1. *Cont.*

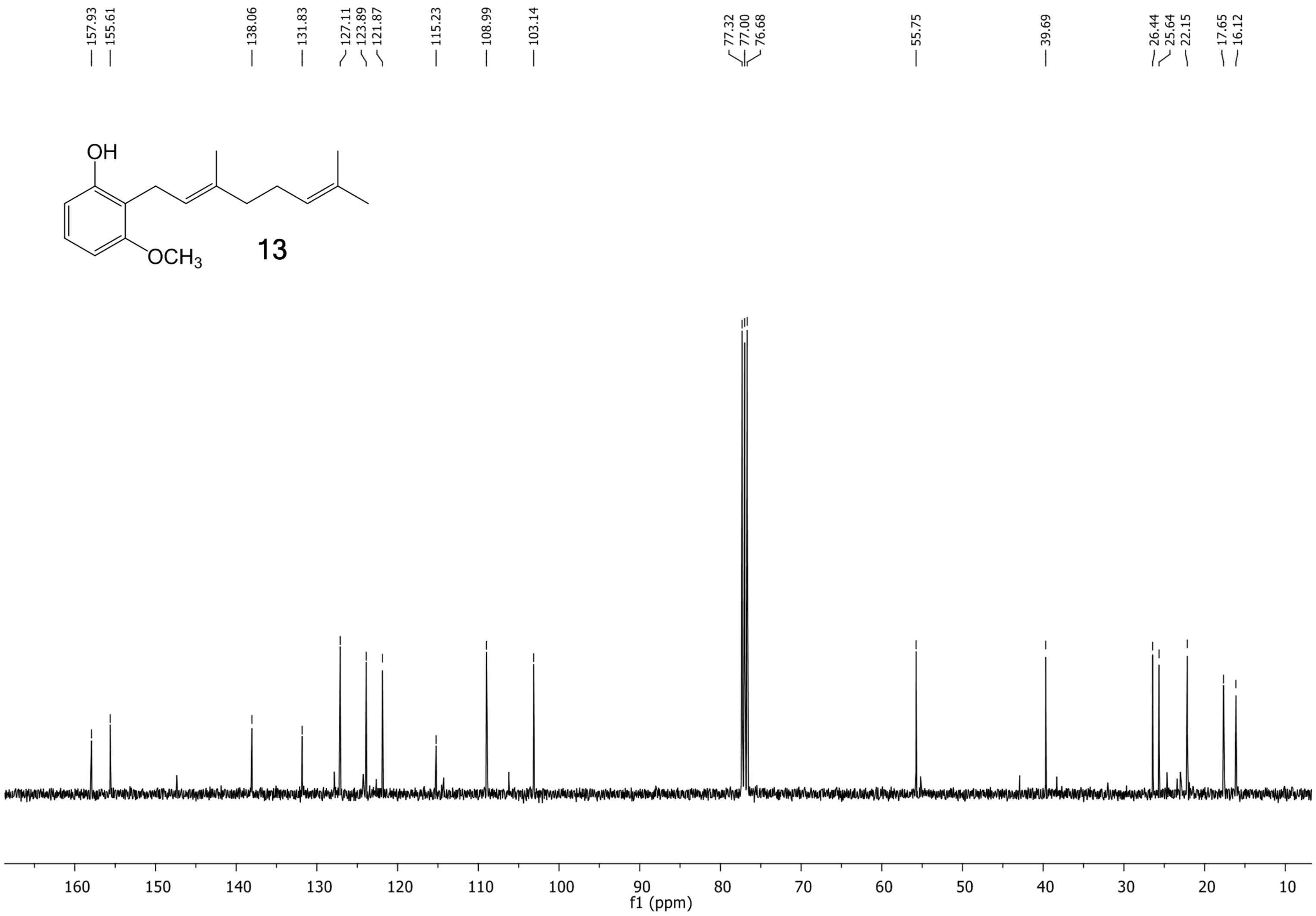

Figure S1. *Cont.*

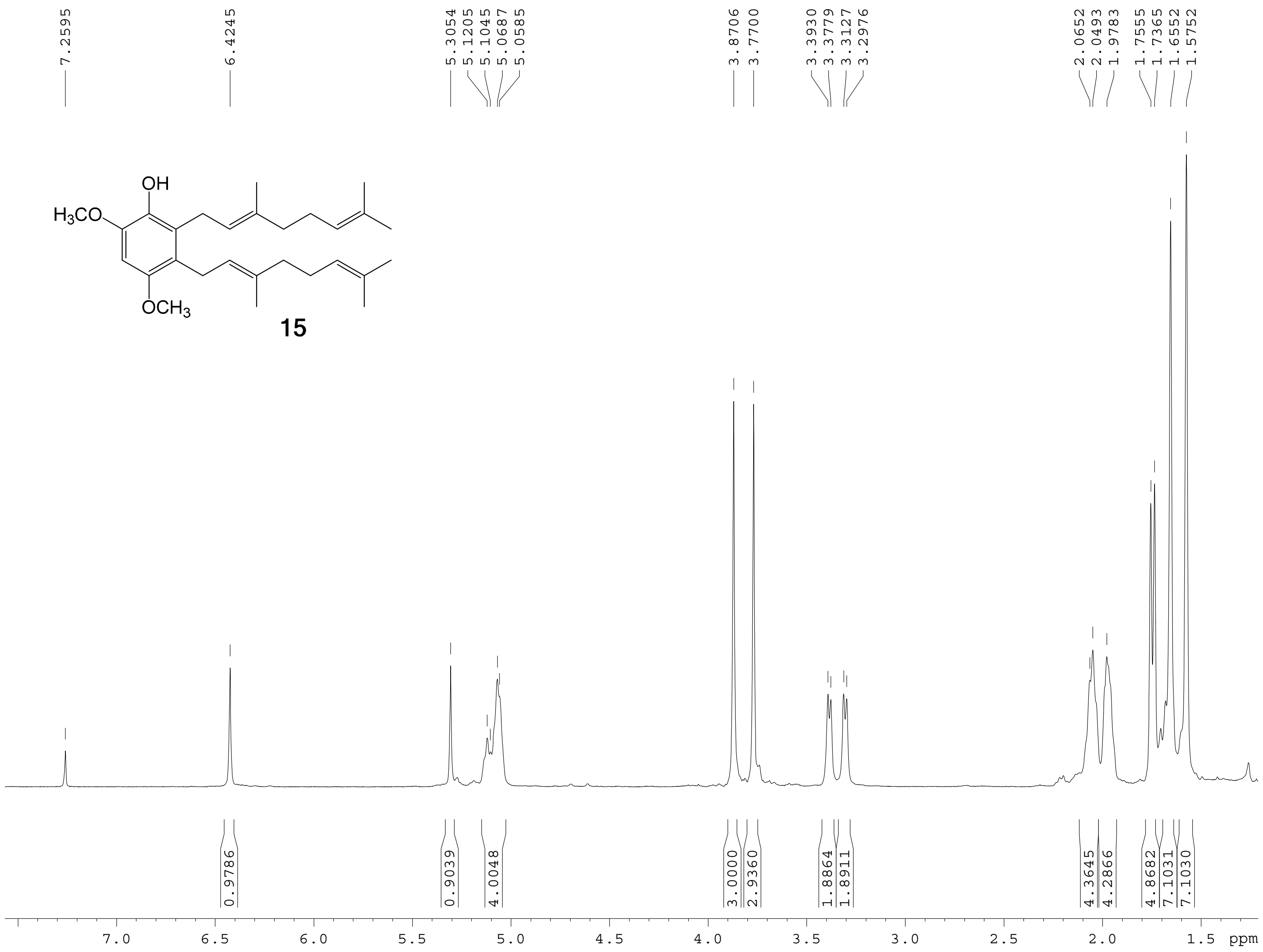

Figure S1. *Cont.*

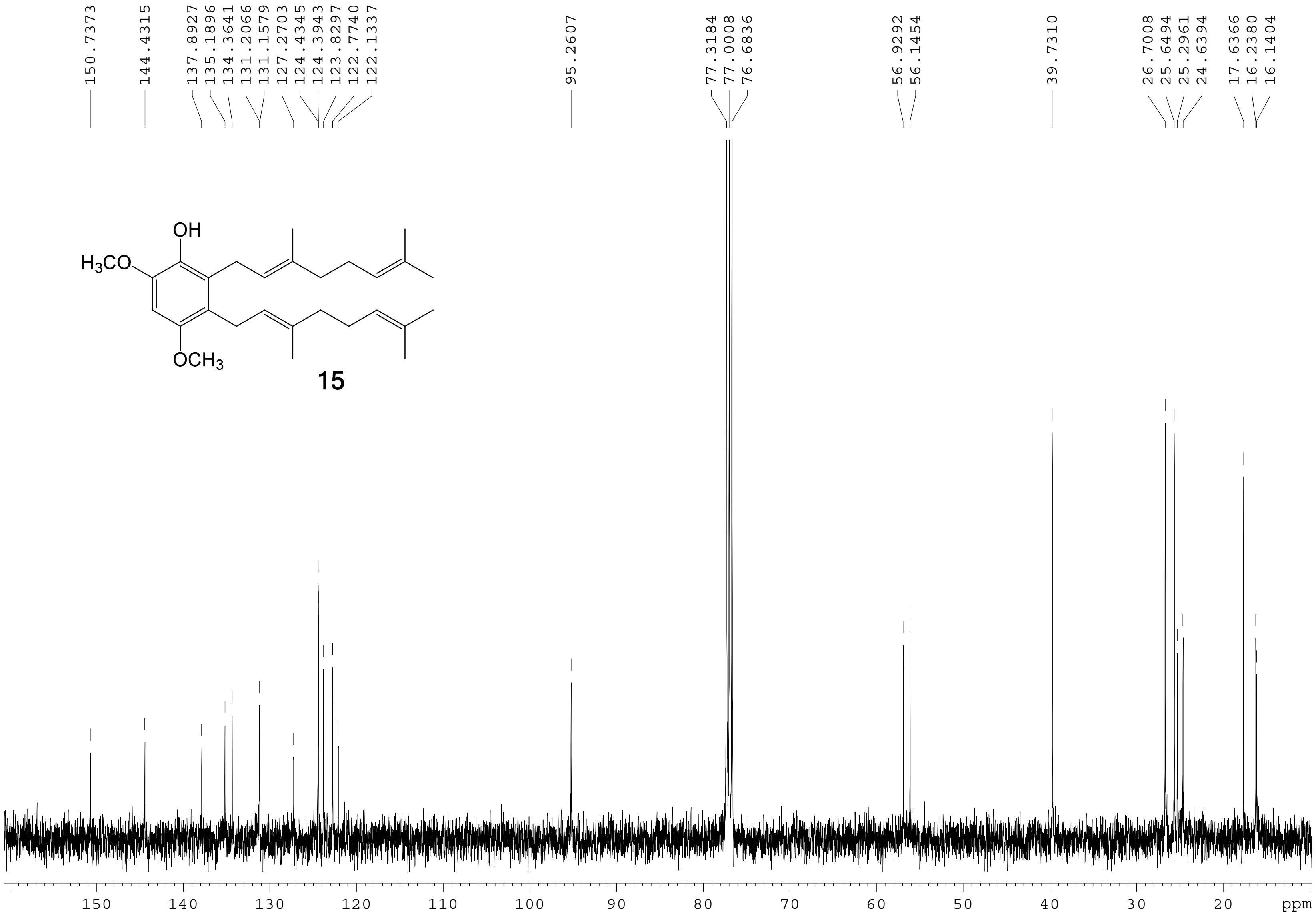

Figure S1. *Cont.*

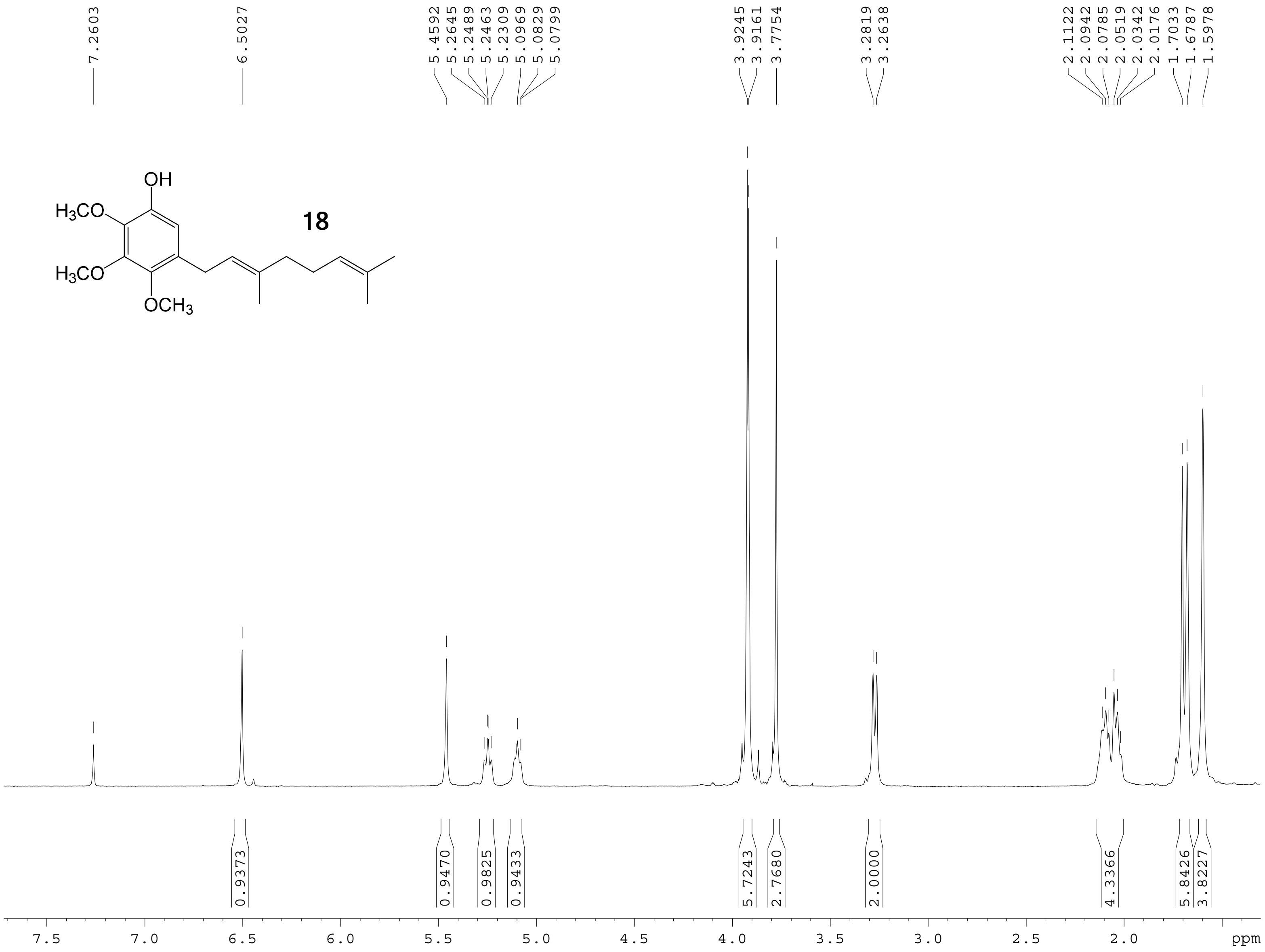

Figure S1. *Cont.*

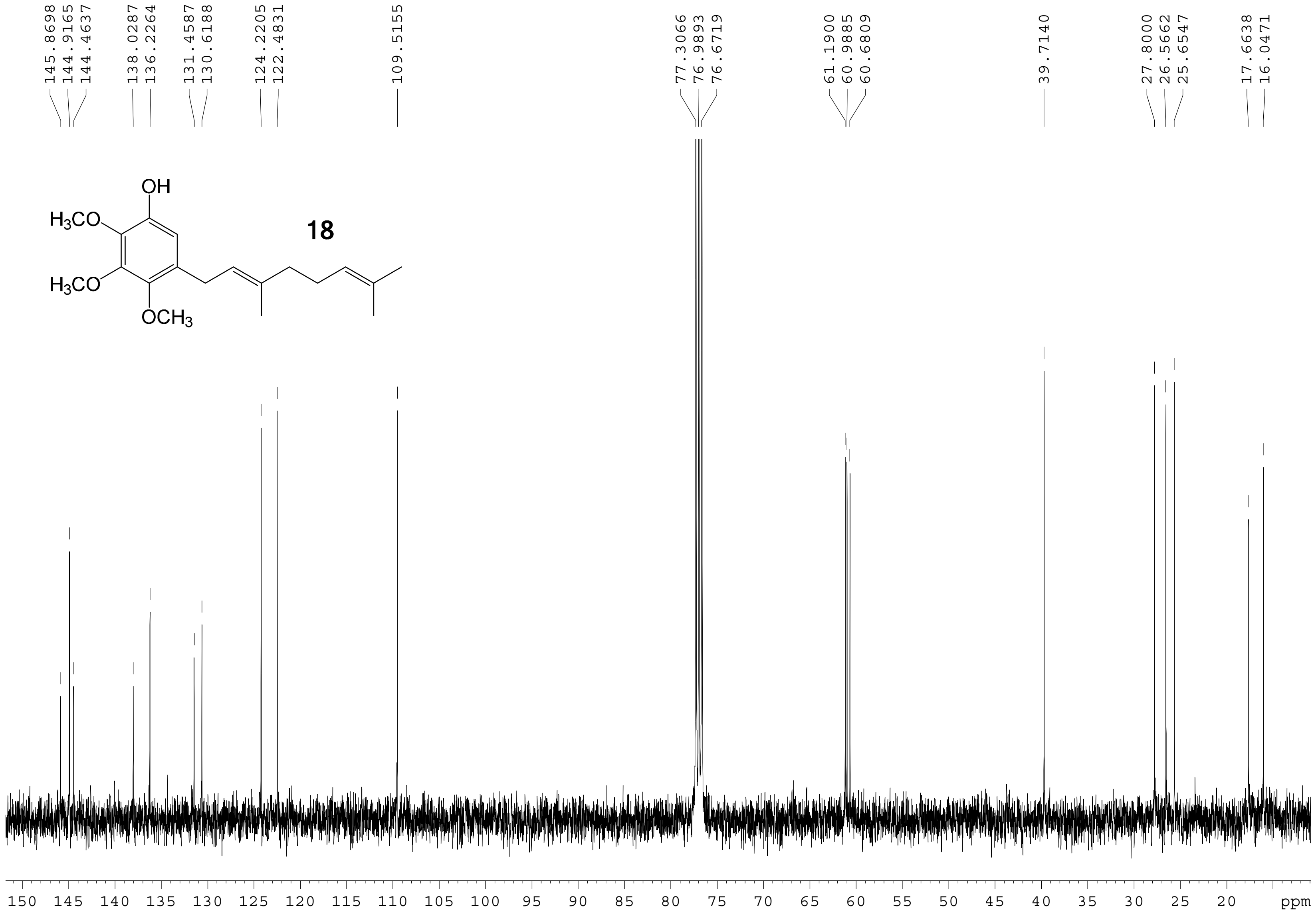

Figure S1. *Cont.*

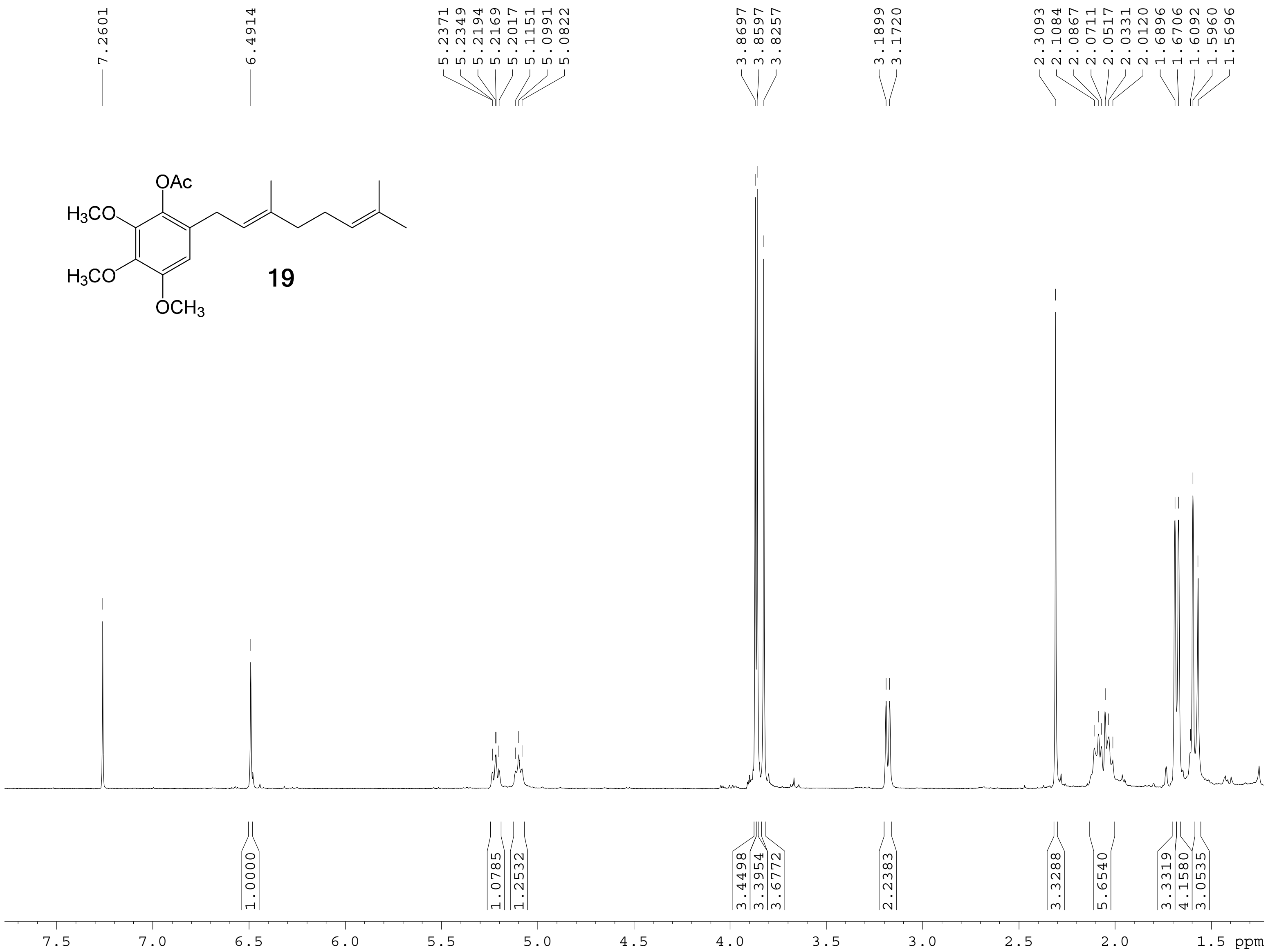

Figure S1. *Cont.*

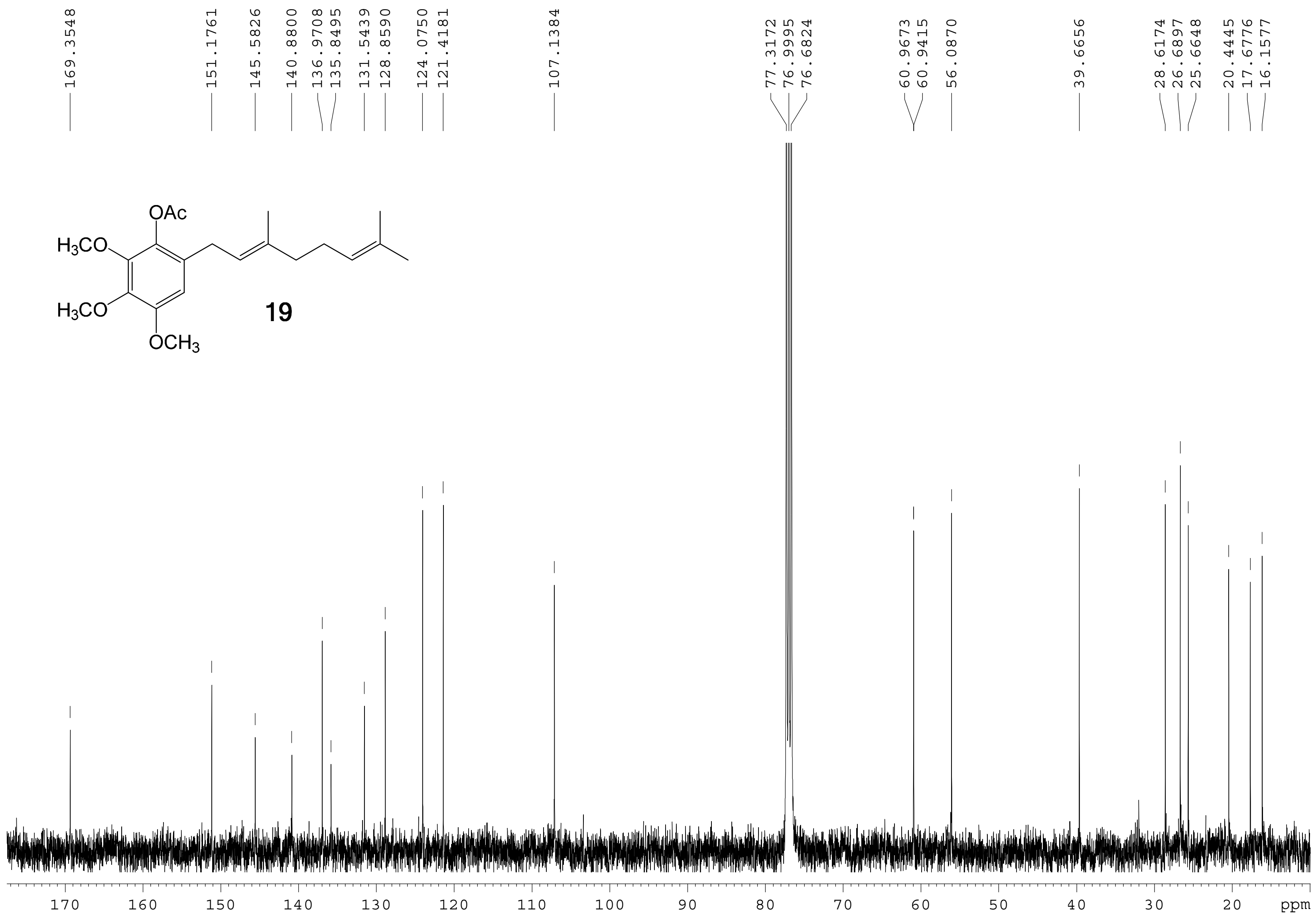

Figure S1. *Cont.*

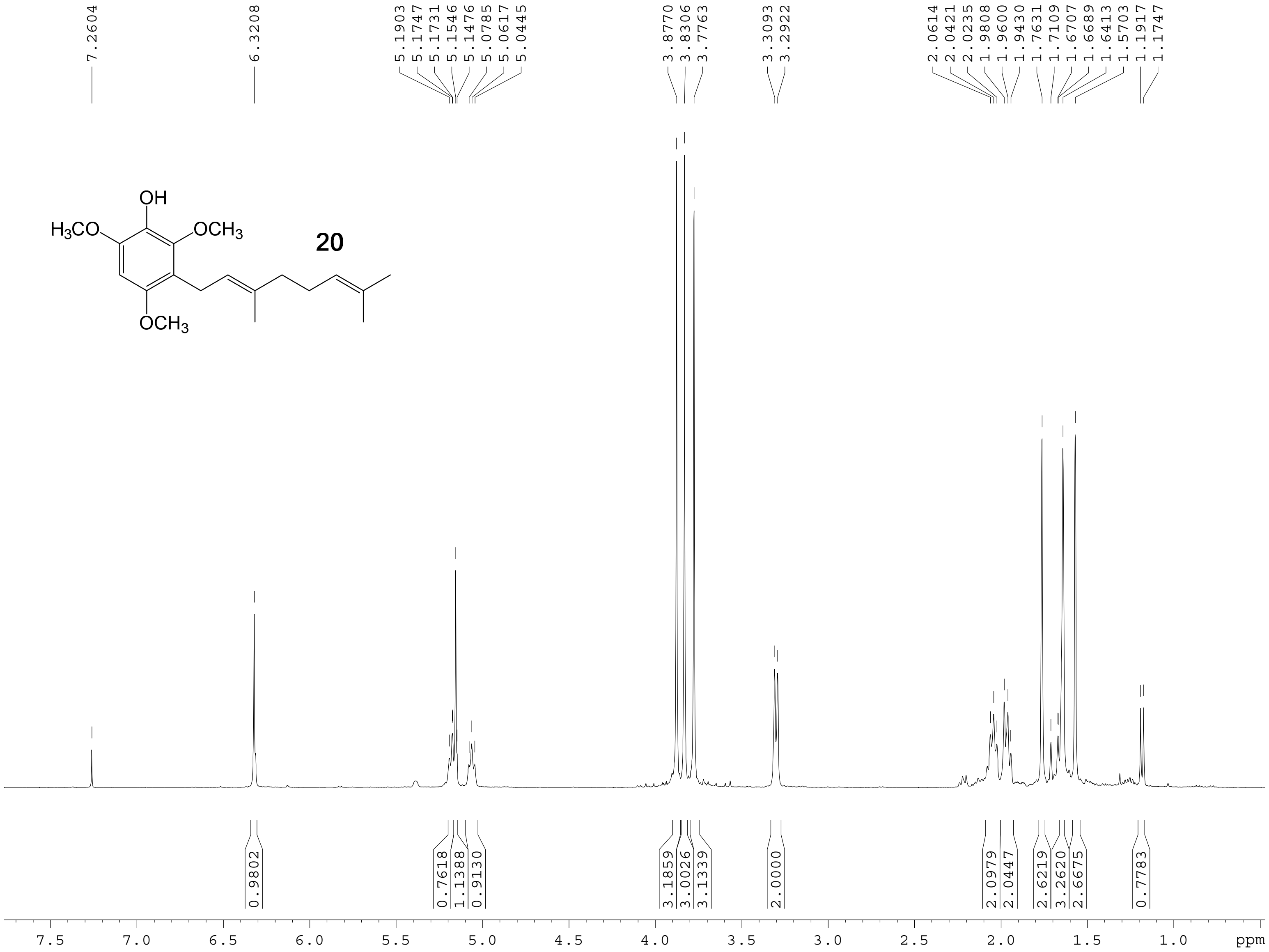

Figure S1. *Cont.*

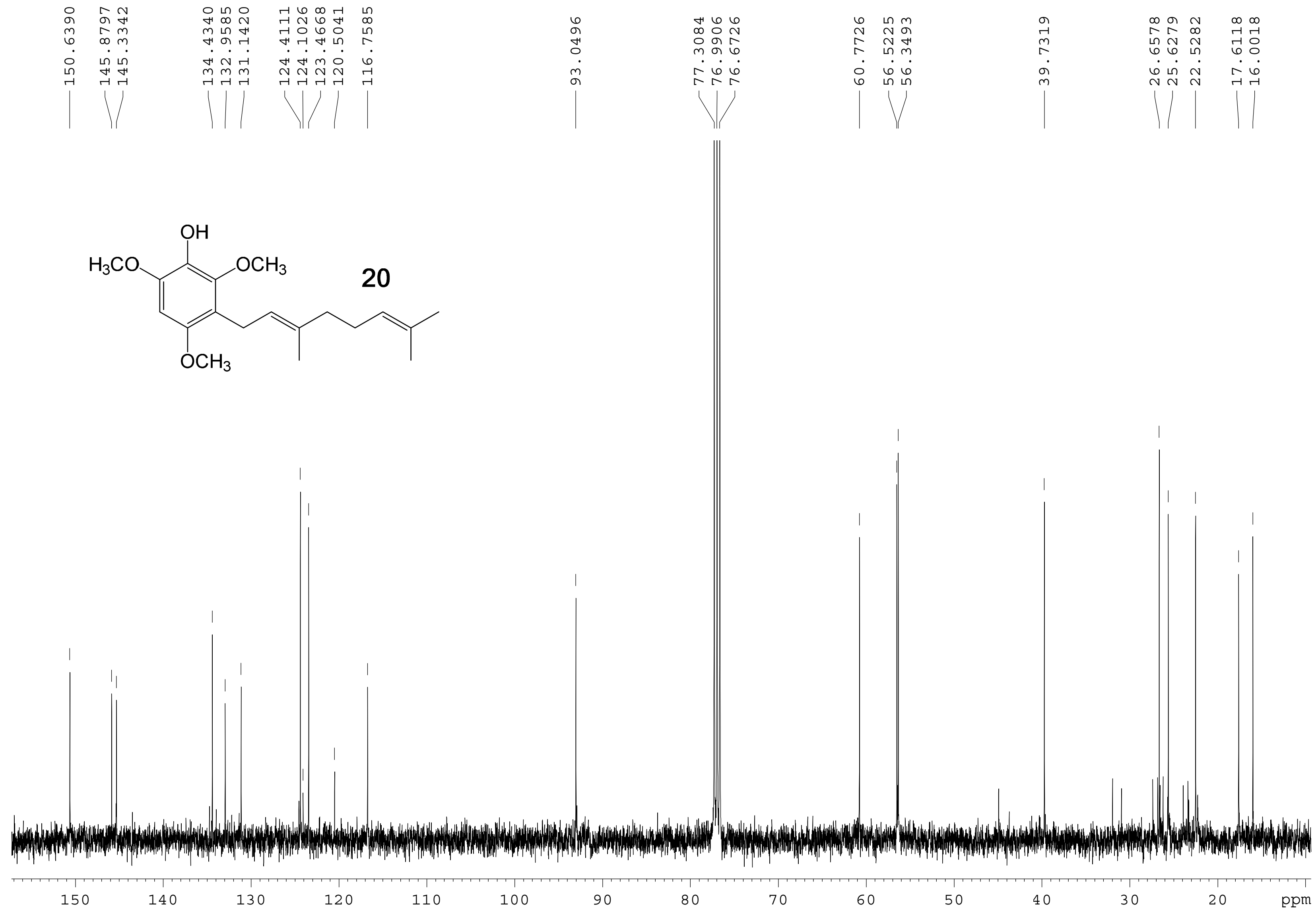

Figure S1. *Cont.*

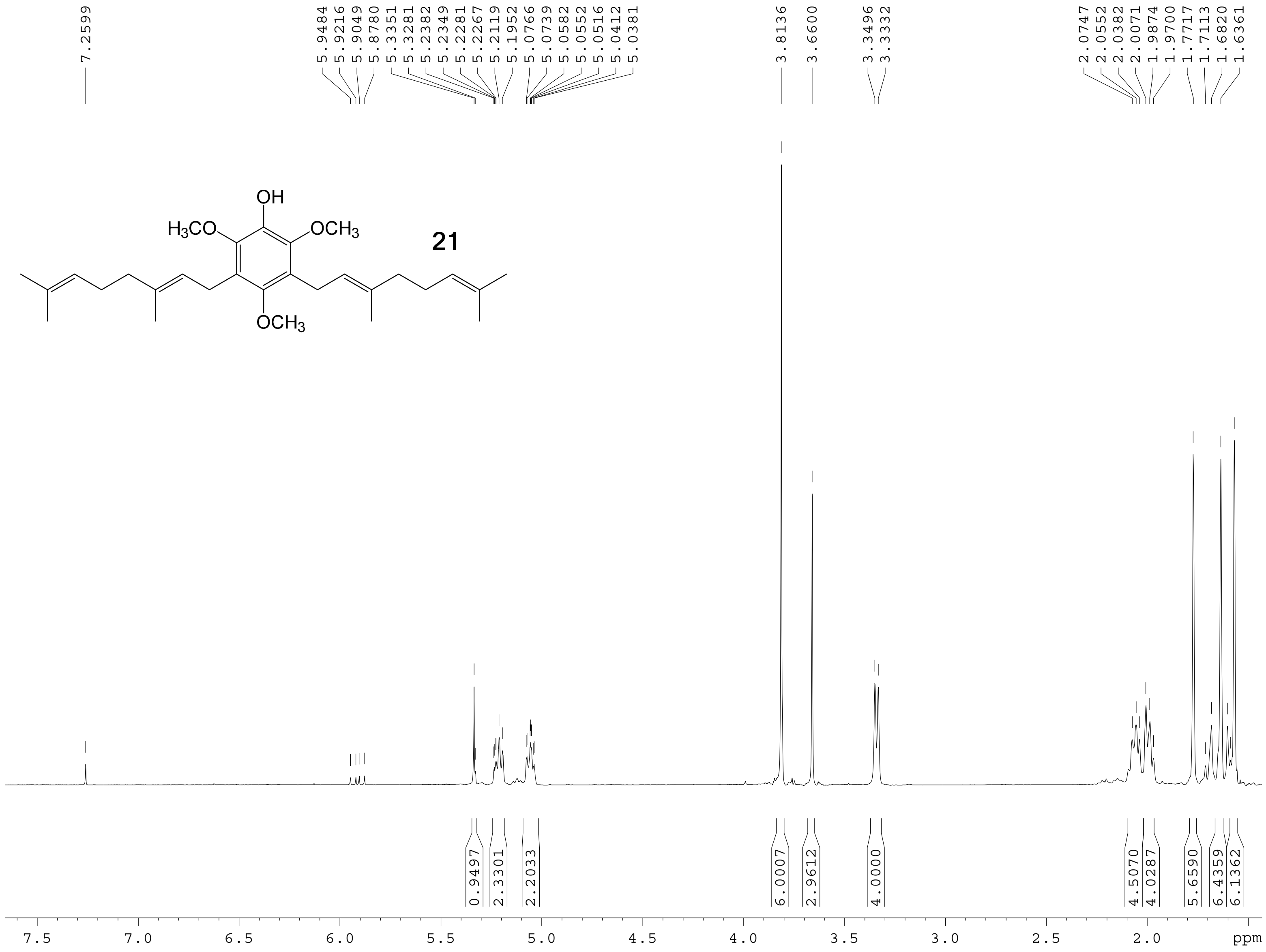

Figure S1. *Cont.*

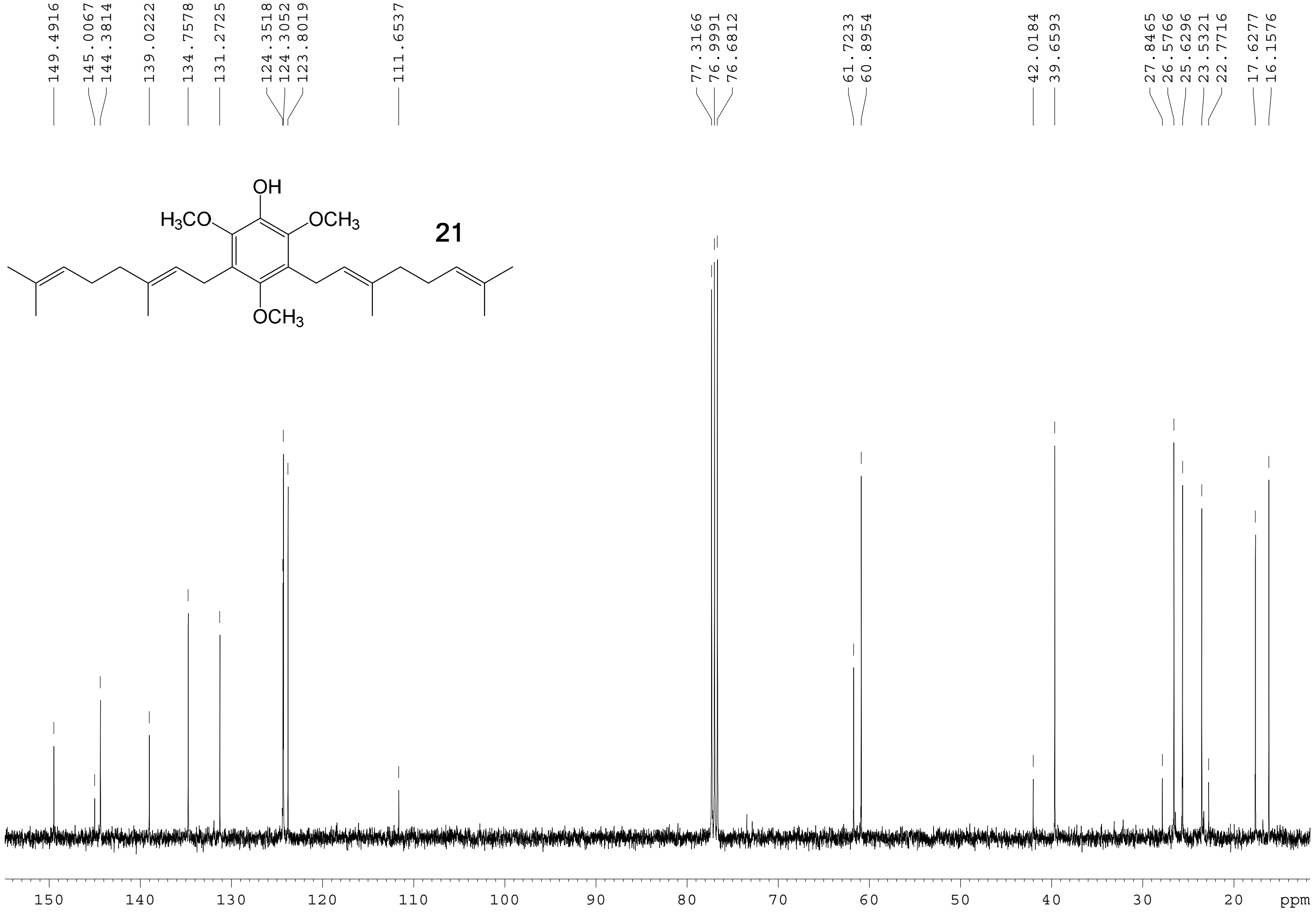

Figure S1. *Cont.*

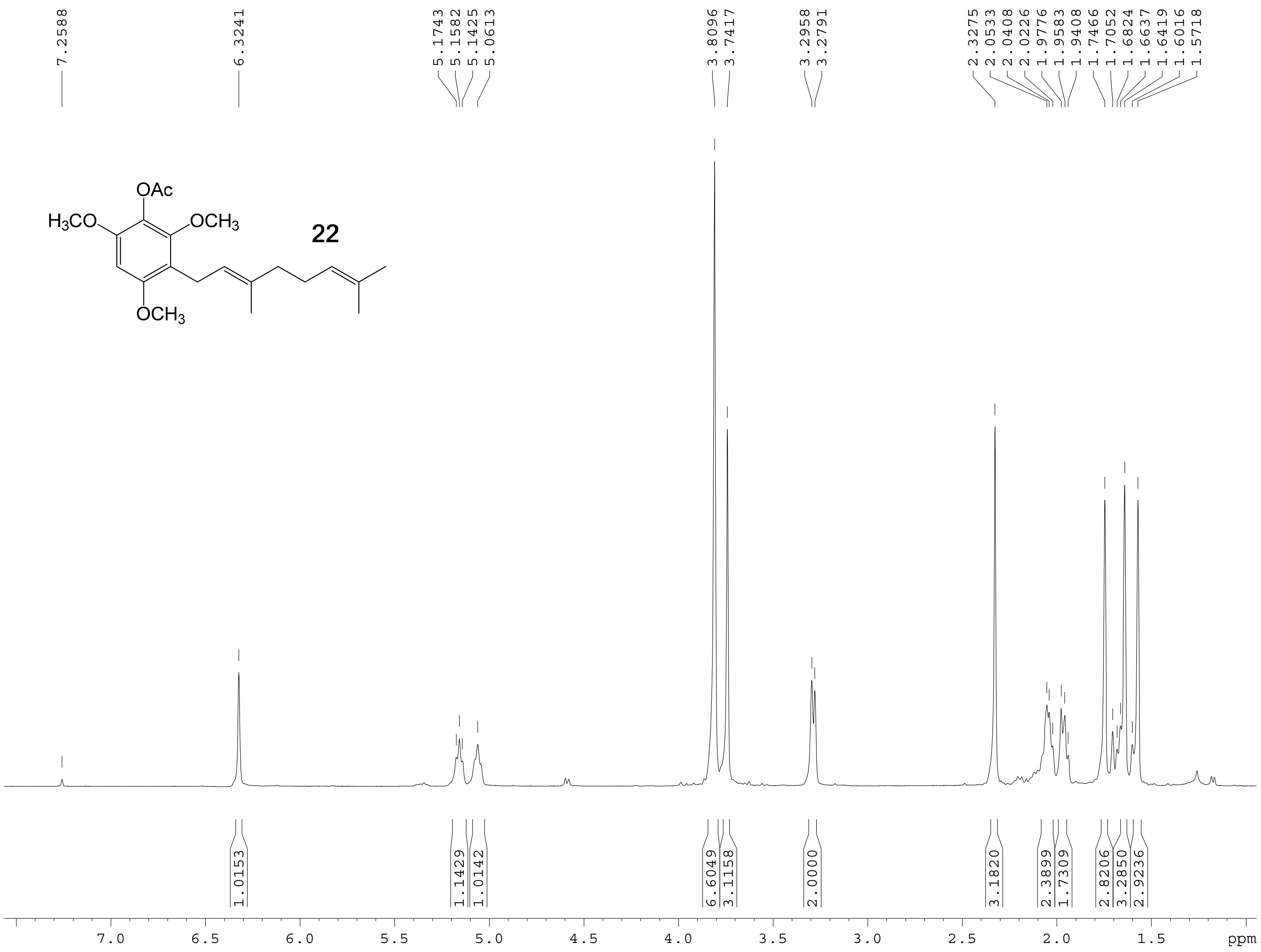

Figure S1. *Cont.*

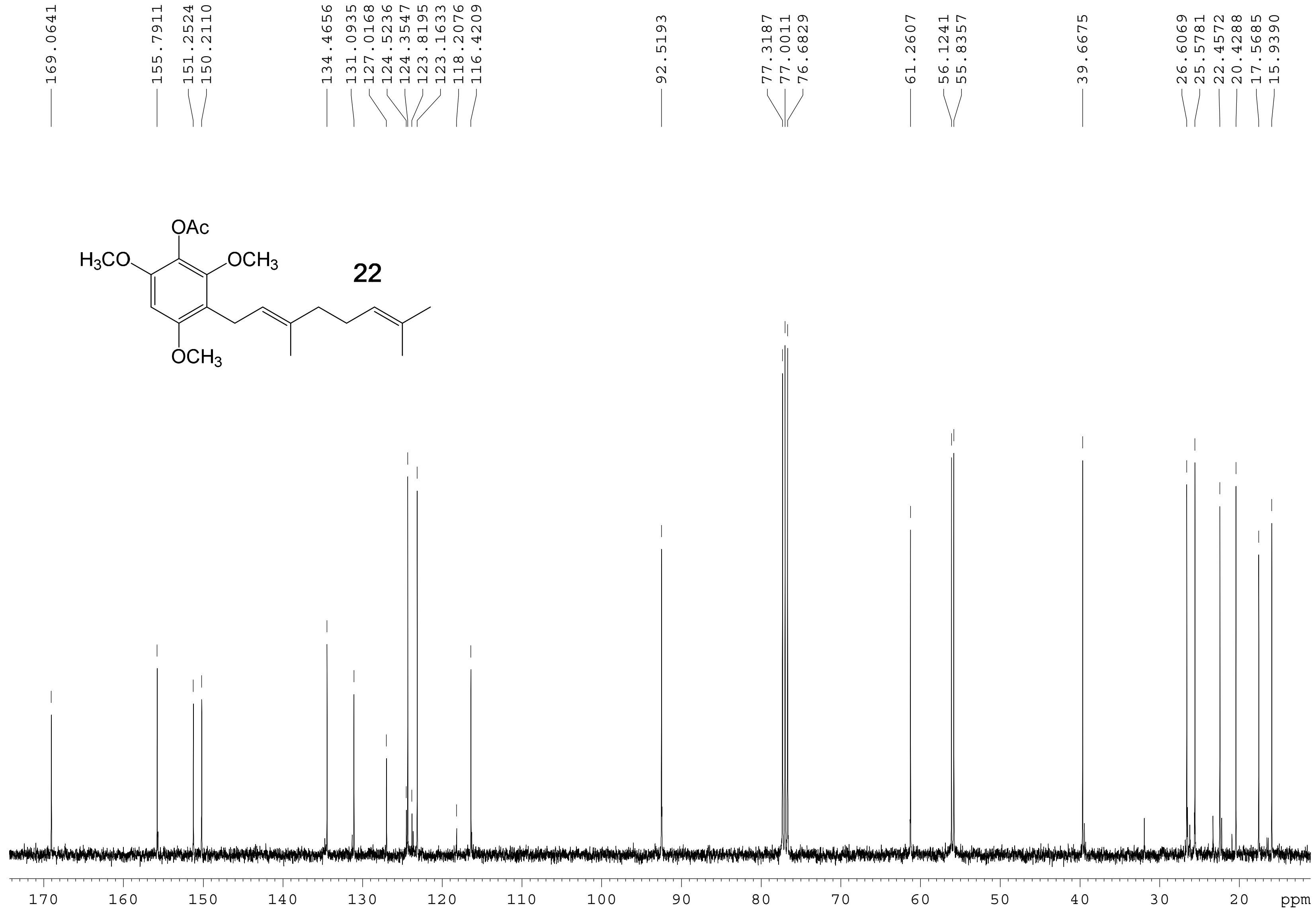

Figure S1. *Cont.*

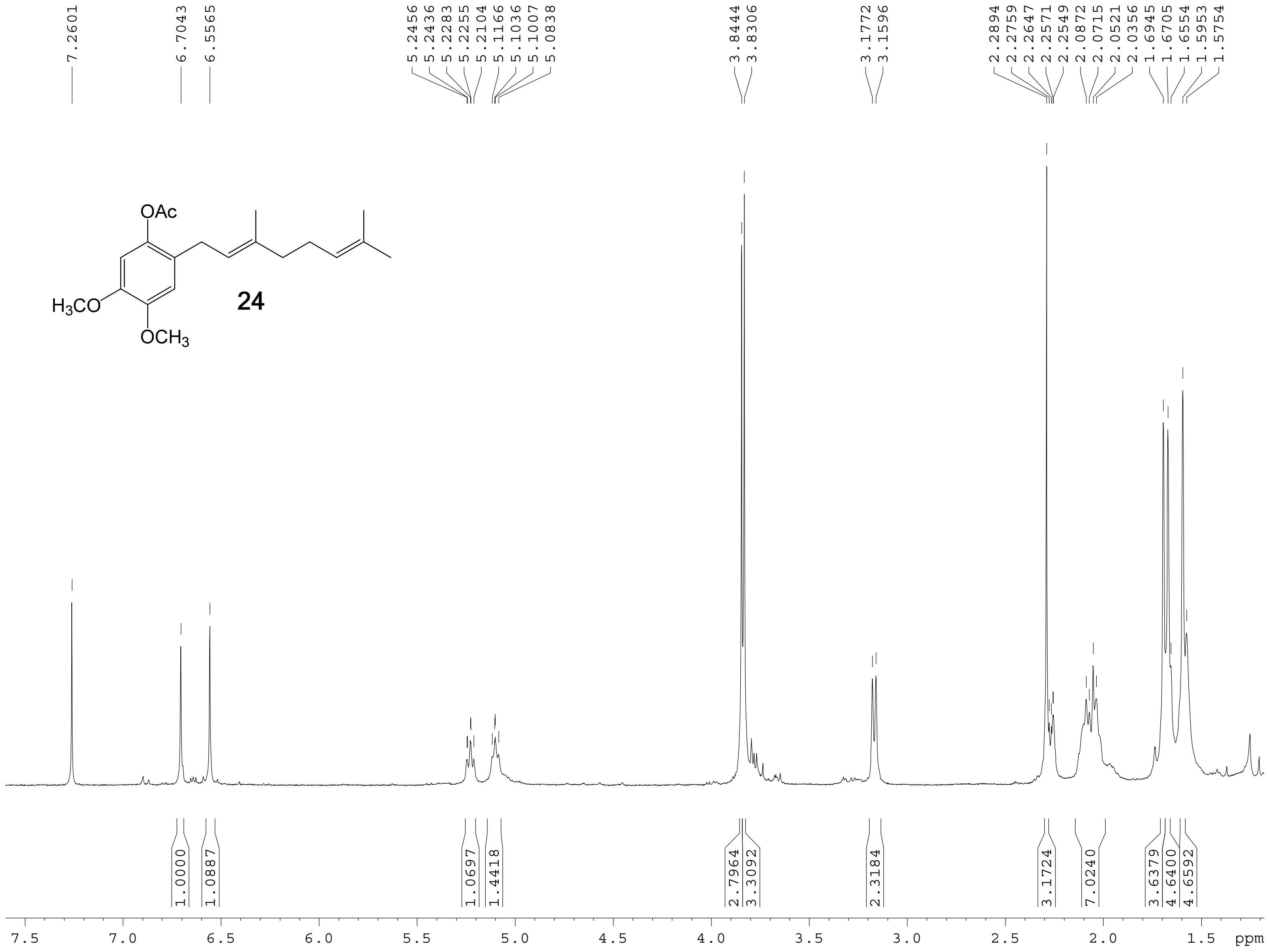

Figure S1. *Cont.*

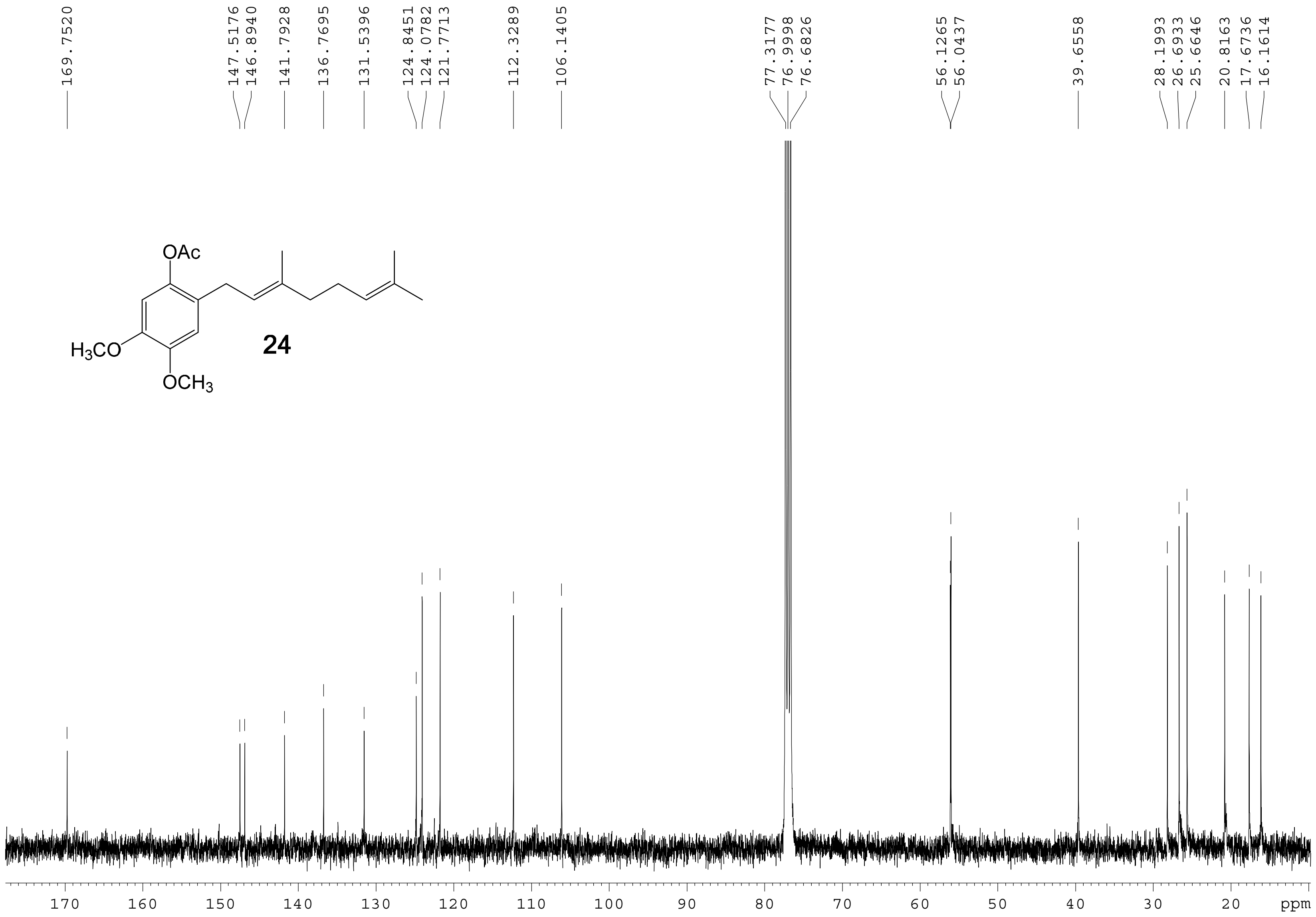

Figure S1. NMR spectra of compounds **12**, **13**, **15**, **18**, **19**, **20**, **21**, **22** and **24**.
